# Three birds with one stone: co-encapsulation of diclofenac and DL-menthol for realizing enhanced energy deposition, glycolysis inhibition and anti-inflammation in HIFU surgery

**DOI:** 10.1186/s12951-022-01437-2

**Published:** 2022-05-06

**Authors:** Haitao Wu, Hu Zhou, Wenjie Zhang, Ping Jin, Qianqian Shi, Zhaohua Miao, Hua Wang, Zhengbao Zha

**Affiliations:** 1 School of Food and Biological Engineering, School of Instrument Science and Opto-Electronics Engineering, Hefei University of Technology, Anhui 230009 Hefei, China; 2 Shenzhen Maternity and Child Healthcare Hospital, The First School of Clinical Medicine, Southern Medical University, Shenzhen, 518028 Guangdong China; 3 Department of Oncology, Inflammation and Immune Mediated Diseases Laboratory of Anhui Province, The First Affiliated Hospital of Anhui Medical University, Hefei, 230022 Anhui China

**Keywords:** High-intensity focused ultrasound, Diclofenac, Phase-change, Glycolysis inhibition, Anti-inflammation

## Abstract

Despite attracting increasing attention in clinic, non-invasive high-intensity focused ultrasound (HIFU) surgery still commonly suffers from tumor recurrence and even matastasis due to the generation of thermo-resistance in non-apoptotic tumor cells and adverse therapy-induced inflammation with enhanced secretion of growth factors in irradiated region. In this work, inspired by the intrinsic property that the expression of thermo-resistant heat shock proteins (HSPs) is highly dependent with adenosine triphosphate (ATP), dual-functionalized diclofenac (DC) with anti-inflammation and glycolysis-inhibition abilities was successfully co-encapsulated with phase-change dl-menthol (DLM) in poly(lactic-co-glycolic acid) nanoparticles (DC/DLM@PLGA NPs) to realize improved HIFU surgery without causing adverse inflammation. Both in vitro and in vivo studies demonstrated the great potential of DC/DLM@PLGA NPs for serving as an efficient synergistic agent for HIFU surgery, which can not only amplify HIFU ablation efficacy through DLM vaporization-induced energy deposition but also simultaneously sensitize tumor cells to hyperthermia by glycolysis inhibition as well as diminished inflammation. Thus, our study provides an efficient strategy for simultaneously improving the curative efficiency and diminishing the harmful inflammatory responses of clinical HIFU surgery.

**Graphical Abstract:**

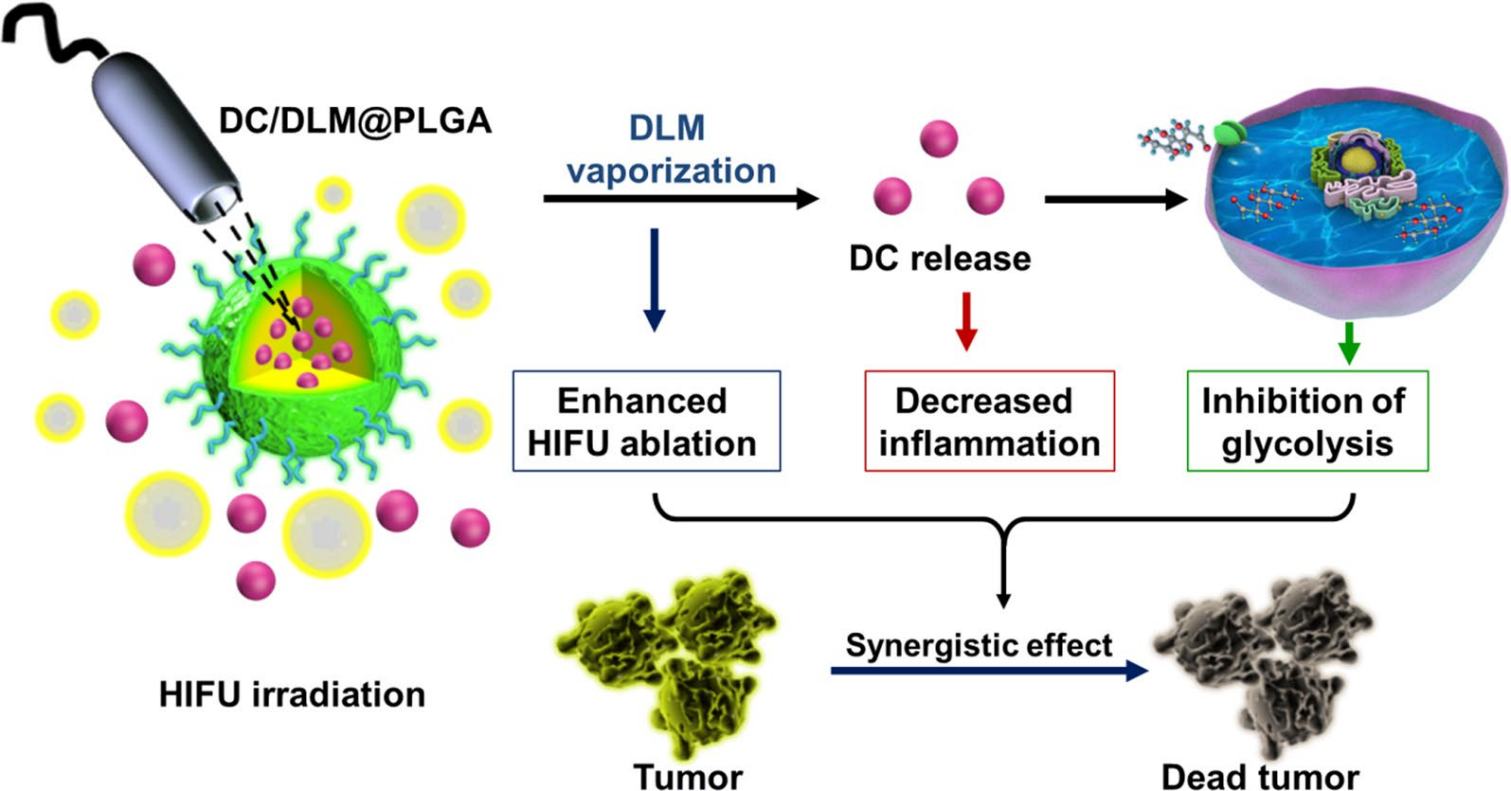

**Supplementary Information:**

The online version contains supplementary material available at 10.1186/s12951-022-01437-2.

## Introduction

Benefiting from the efficient sound-to-heat conversion with outstanding temporal-spatial resolution, high-intensity focused ultrasound (HIFU) surgery is an emerging non-invasive treatment modality for localized tumor therapy [1–4]. Although promising and increasingly used in clinic, the ultrasonic energy for HIFU surgery is inevitably attenuated by the different acoustical impedance of human tissues, which could result in insufficient thermal necrosis from a single HIFU treatment and may lead to a risk of residual tumor [5]. In addition, due to the irregular and ambiguous margins of tumor, ordinarily increasing the energy output of HIFU may also bring the adverse normal tissue injury as well as aggravate the pain of patients [6–11]. Moreover, clinical data have confirmed that tumor cells in the HIFU-irradiated central zones always experience thermal coagulative necrosis accompanied by the release of their intracellular reactive oxygen species and other constituents which would trigger a cascade of inflammatory responses [12]. Although the complex relationship between tumor cells and inflammation-related cytokines in the tumor microenvironment is still not fully understood, the post-treatment inflammation have been recognized as adverse effects which were helpful for stimulating tumor metastasis and recurrence [2, 13–18]. Thus, simultaneously improving the curative efficiency and diminishing the harmful inflammatory responses of HIFU surgery is highly pursued.

Among various sensitized strategies for HIFU surgery, the introduction of synergistic agents (SAs) based on biocompatible micro-/nanoparticles especially with some phase-change species has been demonstrated to be an efficient route for enhancing the energy deposition and ultrasonic cavitation in the desired tissues [19–23]. Despite largely improved ablation outcomes can be obtained, the effectiveness of HIFU enhancement is only one-off due to the complete exhaustion of inner core media once the conventional SAs meet HIFU radiation, which could only realize transient cell ablation. Furthermore, the subsequent adverse harmful inflammatory responses are still in suspense under the utilization of conventional SAs. On the other hand, tumor cells that apart from the HIFU irradiated central zone would commonly activate and up-regulate the expression of intracellular heat shock proteins (HSPs) to establish resistance to heat. As the synthesis of HSPs is highly dependent on adenosine 5′-triphosphate (ATP), blocking the ATP production would be a feasible strategy for weakening the heat-resistance of tumor cells. In comparison to normal tissues, tumor cells are generally characterized with over-expressed glucose transport proteins (Gluts) to uptake much more glucose for rapid growth through energy-inefficient aerobic glycolysis [24, 25]. Therefore, it is logical that inhibiting the overexpression of Gluts in tumor cell membranes would reduce the intracellular uptake of glucose and the production of ATP [26–31], which would be beneficial for lowering the expression of HSPs and then sensitizing tumor cells to HIFU surgery.

**Scheme 1:**
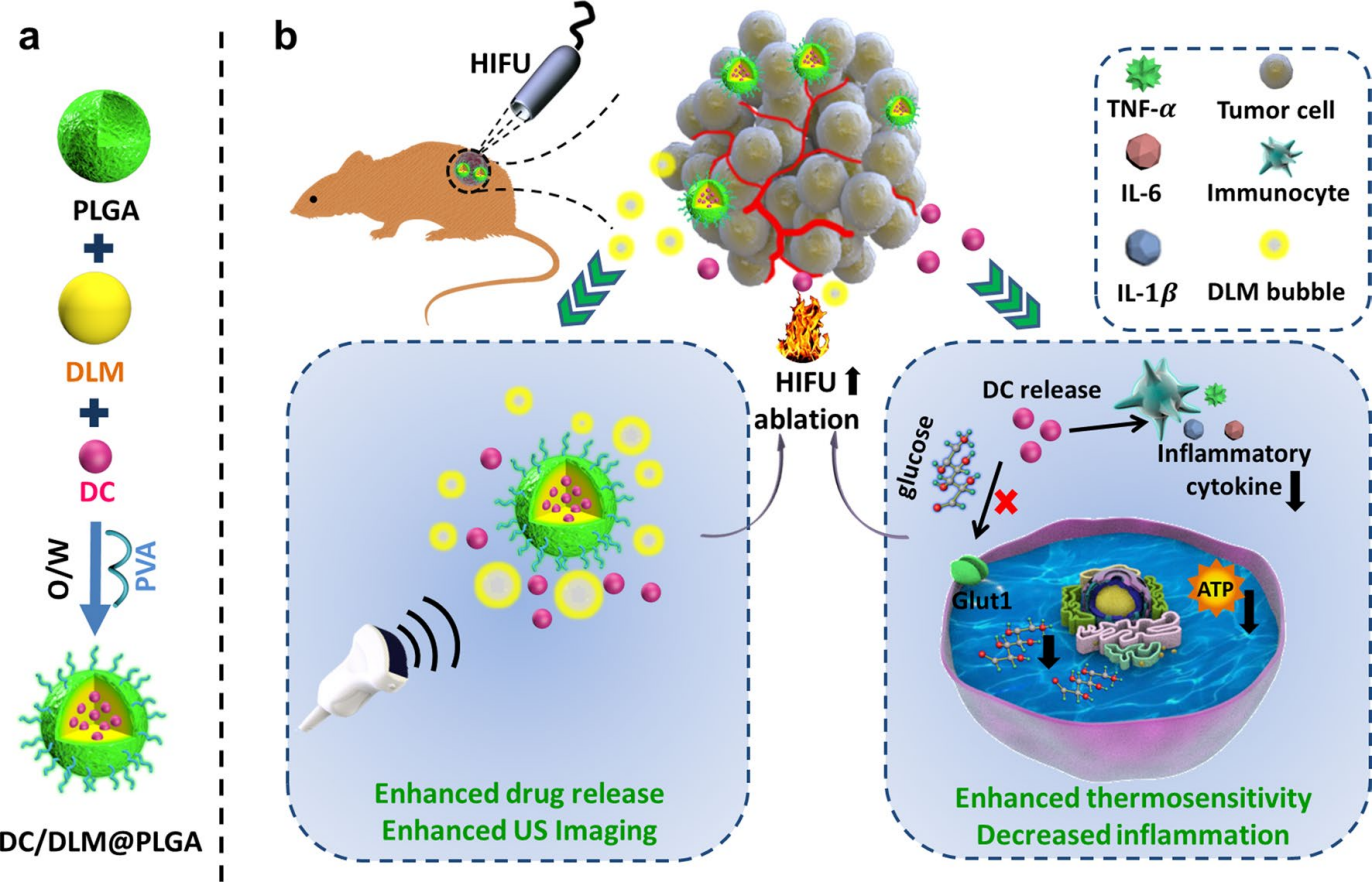
Schematic illustration of sensitizing tumor cells to HIFU by tumor glycolysis inhibition via DC/DLM@PLGA NPs. **a** The synthesis process of PLGA NPs encapsulating DLM and DC; **b** principles of enhancing HIFU-mediated anti-tumor efficacy and decreased inflammation

In this work, diclofenac (DC, a commonly used anti-inflammatory drug in clinic and small molecule inhibitor with high selectivity toward Glut1[32]) and dl-menthol (DLM, a natural phase-change medium) were co-encapsulated in poly(lactic-co-glycolic acid) (PLGA) nanoparticles (DC/DLM@PLGA NPs) *via* the typical oil-in-water emulsion method for simultaneously improving the curative efficiency and diminishing the harmful inflammatory responses of HIFU surgery (Scheme 1a). Once the as-prepared DC/DLM@PLGA NPs accumulated in the tumor region, the thermal effect of HIFU irradiation would induce the phase transition of DLM, which could change the acoustic environment of the tumor site to enhance the ablation effect of HIFU surgery. Subsequently, the released DC would result in down-regulation of Glut1 and consequently inhibit glucose metabolism as well as ATP-dependent HSPs synthesis. Meanwhile, the anti-inflammatory effect of DC reduced the adverse inflammatory responses after HIFU surgery (Scheme 1b).

## Result and discussion

**Fig. 1:**
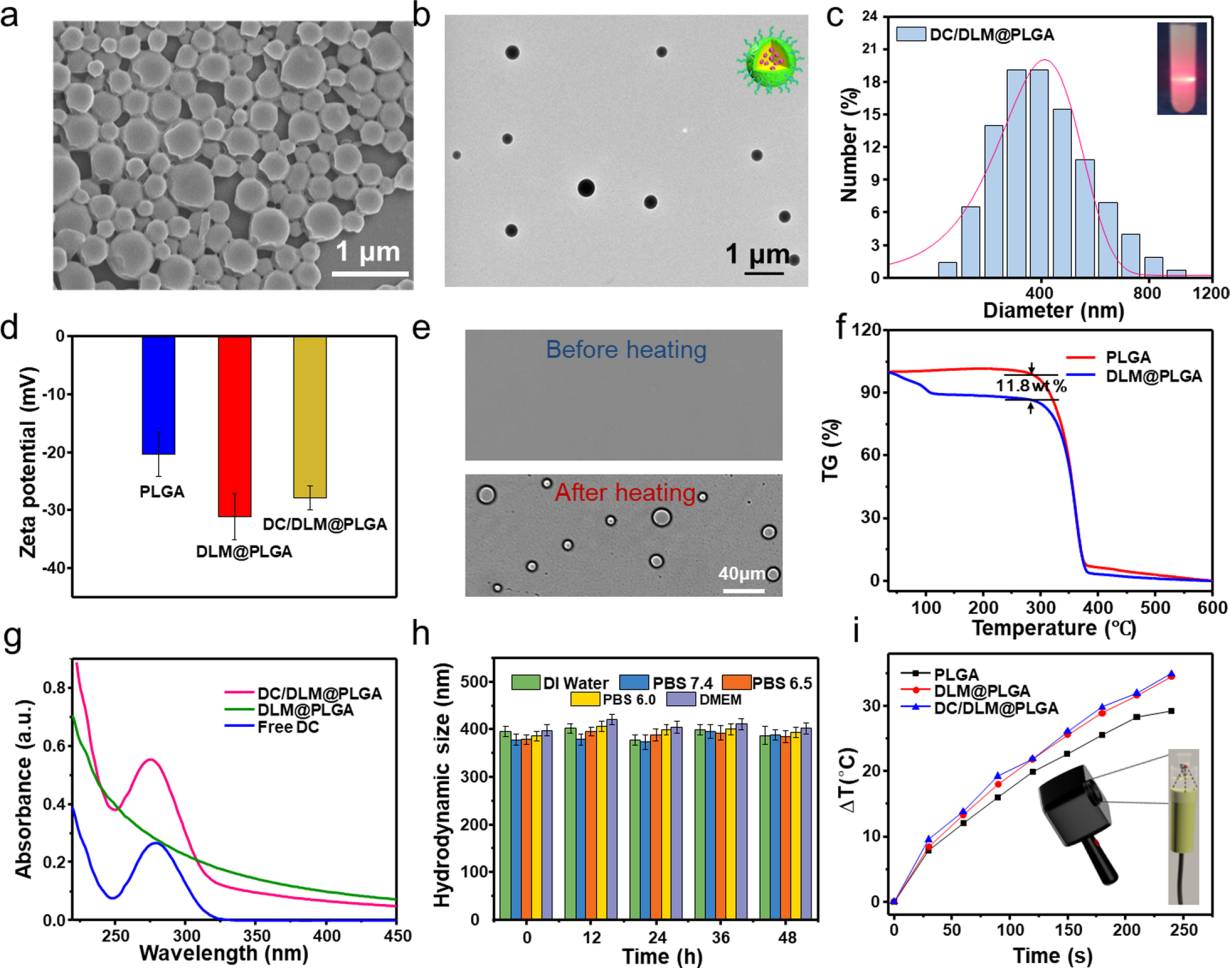
**a** SEM and **b** TEM images of DC/DLM@PLGA NPs. **c** hydrodynamic diameter distribution (inset: Tyndall effect of DC/DLM@PLGA NPs) and **d** zeta potential. **e** Microscopy images of DC/DLM@PLGA NPs under heating for 60 s. **f** TG curve of DC/DLM@PLGA NPs and **g** UV-vis spectra. **h** Stability of DC/DLM@PLGA NPs in various media. **i** Temperature elevation curves of different NPs under HIFU irradiation (power: 25 W; duty cycle: 50%; 3 s on and 3 s off). (Inset: experimental setup for monitoring temperature change by an infrared camera)

SEM and TEM (Fig. 1a, b) were firstly used to confirm that the as-prepared DC/DLM@PLGA NPs were successfully developed with a relatively uniform spherical morphology. Due to the existence of PVA molecules on their surfaces, the DC/DLM@PLGA NPs were homogeneous dispersed which could be confirmed by the typical Tyndall phenomenon as well as an average hydrodynamic diameter of around 400 nm (Fig. 1c) and negatively charged with a zeta potential of − 27.9 mV (Fig. 1d), respectively. To prove the successful loading of volatile DLM, the aqueous solution of obtained nanoparticles was firstly heated from room temperature to 60 °C (the strong volatility of DLM render them the ability to bubbling at this temperature) and then the bubble-generation performance was checked by an inverted fluorescence microscope. As shown in Fig. 1e, the appearance of many bubbles after heating at 60 °C was ascribed to the liquid-gas phase transition of DLM, indicating its successful encapsulation in as-prepared NPs which was further confirmed by the result of thermogravimetric analysis showed that the loading content of DLM in DLM@PLGA NPs was approximately 11.8 wt% (Fig. 1f). Benefiting from the characterized UV-vis absorption spectrum of DC, the appearance of an absorption peak at 277 nm demonstrated the successful encapsulation of DC in obtained DC/DLM@PLGA NPs (Fig. 1g), and the loading content of DC was calculated approximately as 10.6% (Additional file 1: Figure S1). Furthermore, the as-prepared DC/DLM@PLGA NPs exhibited good dispersity and stability in various media without obvious size change or macroscopic aggregates (Fig. 1 h, Additional file 1: Figure S2).

Next, the in vitro synergistic effect of DLM was investigated by using the established experimental platform (Additional file 1: Figure S3). As shown in Additional file 1: Figure S4, the temperature elevation of PBS solution under HIFU irradiation showed a power-dependent manner and the result demonstrated that the phase transition temperature of DLM could be easily achieved under HIFU irradiation with power input of 25 W for less than 2 min. Without the existence of DLM, the temperature elevation of PLGA aqueous solution (10 mg/mL, 600 µL) could achieve 29.2 °C under 4 min HIFU irradiation (25 W), while the DLM@PLGA and DC/DLM@PLGA solution increased by 34.5 and 34.9 °C, respectively (Fig. 1i; Additional file 1: Figure S5). This DLM-enhanced temperature elevation could be attributed to the gasified DLM microbubbles which would intercept partial energy of HIFU, reducing the energy loss to the outside and enhancing the acoustic cavitation effect of the ultrasound [33].

**Fig. 2:**
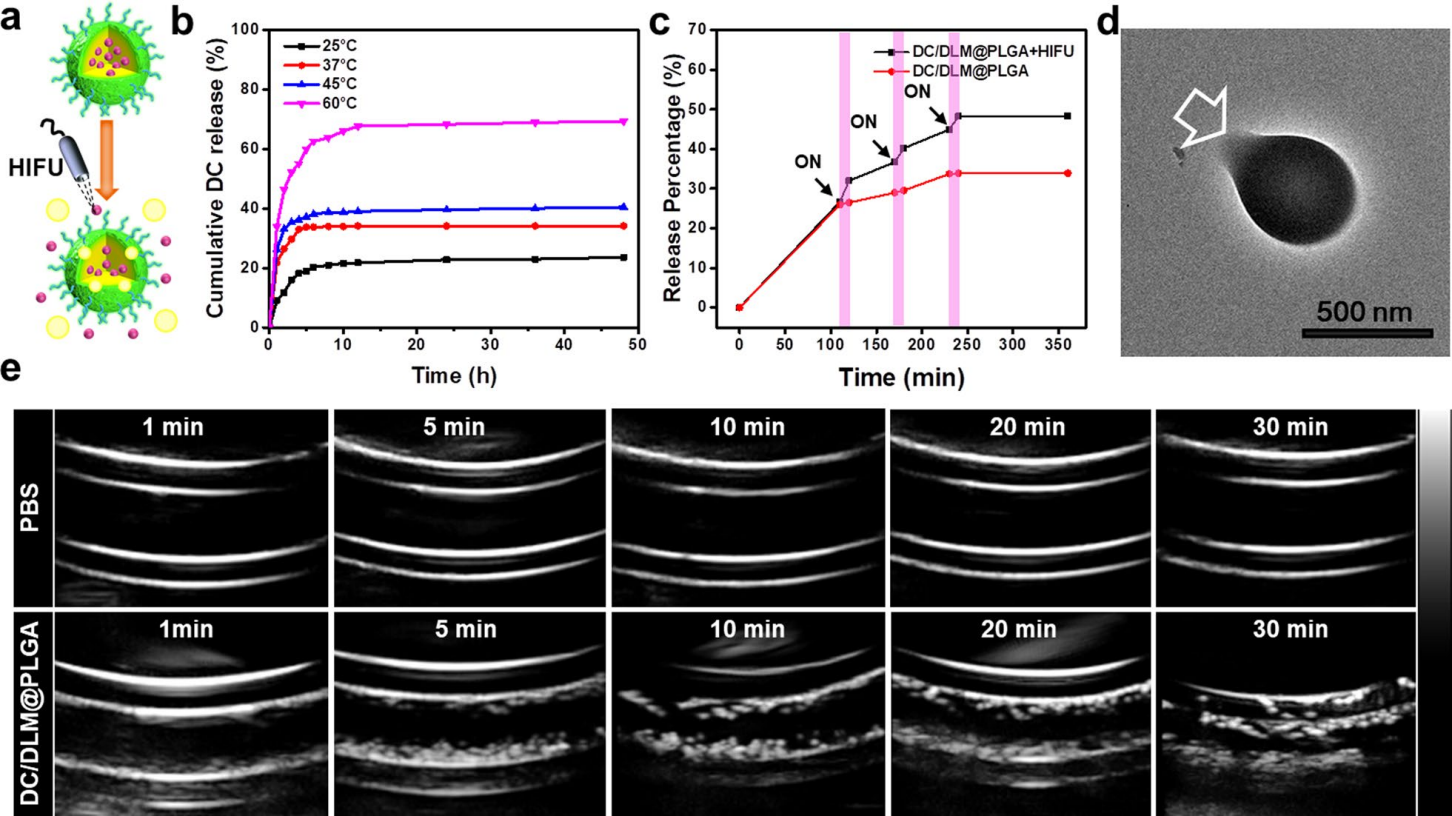
**a** Schematic illustration of DC release from DC/DLM@PLGA NPs under HIFU irradiation. **b** DC release profiles under different temperatures and **c** DC release performances from PLGA NPs triggered by HIFU irradiation. **d** TEM image of typical DC/DLM@PLGA NPs after HIFU irradiation. **e** Sustained ultrasonic contrast images of DC/DLM@PLGA NPs (10 mg/mL, 3 mL) in a 60 °C water bath

Due to the main driven force for DC release was from solid–liquid–gas tri-phase transition of DLM (Fig. 2a), the in vitro DC release behaviors were then first investigated at various temperatures. As shown in Fig. 2b, the amount of released DC was positively correlated with solution temperature. For instance, only 23.6% of DC (these DC molecules were speculated to be encapsulated in PLGA network) was released from the DC/DLM@PLGA NPs within 48 h when the solution temperature was lower than the melting point of DLM. Once the temperature exceeds the melting point of DLM, the amount of released DC increased rapidly. Particularly, due to the strong volatility of DLM and bubbling ability, heating the aqueous solution of DC/DLM@PLGA NPs to 60 °C could result in approximately 69.3% of DC release within 12 h. Inspired from the thermo-sensitive DC release profiles, HIFU, as a targeted heat source, was then expected to be appropriate for inducing more controllable drug release as previously reported [34]. With controlled HIFU irradiation for three rounds (5 min each round, power: 25 W; duty cycle: 50%; 3 s on and 3 s off), an enhancement of around 15% of DC release was successfully achieved by the volatilization of DLM caused by HIFU-generated heat and the mechanical effect of HIFU (Fig. 2c) [35], further confirmed by the structure destruction of DC/DLM@PLGA NPs (Fig. 2d). In addition, the bubbling performance of DC/DLM@PLGA NPs under 60 °C water bath was further confirmed by naked eyes and monitored by a clinical ultrasound imaging system (Sonix-Touch). Satisfactorily, sustained acoustic signal increments in the in vitro contrast ultrasound image of DC/DLM@PLGA NPs, indicating their good potential for real-time monitoring and programmed DC release (Fig. 2e, Additional file 1: Figure S6).

**Fig. 3:**
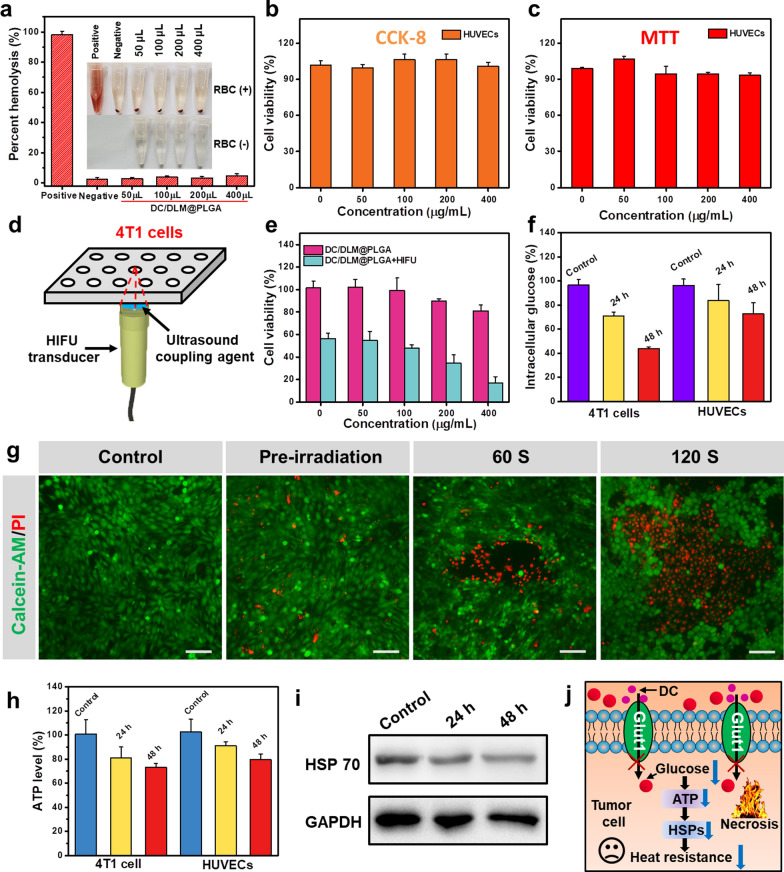
**a** Hemolysis test of DC/DLM@PLGA NPs at different concentrations. **b** MTT and **c** CCK-8 assays. **d** Schematic diagram of experimental setup for 4T1 cells exposed to 50% duty cycle HIFU at 25 W. **e** Cytotoxicity of different DC/DLM@PLGA NPs with gradient concentrations. **f** Intracellular glucose contents of 4T1 cells and HUVECs after co-incubation with DC/DLM@PLGA NPs for 24 or 48 h. **g** Live/Dead fluorescent staining of 4T1 cells in PBS, DC/DLM@PLGA NPs, and DC/DLM@PLGA NPs + HIFU groups (60 and 120 s), Scale bar: 100 μm. **h** Intracellular ATP content measurement and **i** analysis of HSPs expression after treatment with DC/DLM@PLGA NPs, untreated cell served as a control group. **j** Schematic representation of the process of DC-induced reduction in protein synthesis

The biocompatibility of nanomaterials is a crucial factor that should be firstly considered for potential clinical applications. Following the typical protocol of hemolytic assay, the hemolysis rate of as-prepared DC/DLM@PLGA NPs was determined to be around 4.6% (lower than the threshold value of 5%) even at a dose as high as 800 µg/mL, suggesting the NPs would be relatively safe in the blood circulation (Fig. 3a). Consistent result was obtained by co-incubation of HUVECs (used here as a normal cell line) with gradient concentrations of NPs. As shown in Fig. 3b and c, both standard MTT and CCK-8 assays demonstrated the good cytobiocompatibility of DC/DLM@PLGA NPs as other reported PLGA-based nanoplatform [36, 37]. As the generation of HSPs is highly ATP-dependent, the Glut1 inhibiting property of DC has been reported to be a potential strategy for weakening the glucose metabolism and thus reducing the expressed ATP levels [38]. This inhibitory effect was more obvious on tumor cells with over-expressed Glut1 molecules, which was also consistent with cell-proliferation inhibiting effect of DC confirmed by the typical MTT assay (Additional file 1: Figure S7) [39]. Therefore, the in vitro synergistic effect from HIFU-induced hyperthermia and glucose metabolism inhibition *via* DC was then investigated by a self-made experimental set-up (Fig. 3d). In comparison to reserved 80% cell viability of DC/DLM@PLGA treated only group, a much more cell killing effect was obtained for DC/DLM@PLGA NPs group under HIFU irradiation (Fig. 3e). In comparison to the DLM@PLGA NPs treated group, the intracellular glucose content in 4T1 tumor cells decreased to 70.9 and 44% after treatment with DC/DLM@PLGA NPs for 24 and 48 h, respectively, which was also lower than the glucose content in HUVECs (Fig. 3f). In addition, the synergistic anti-tumor effect of HIFU surgery and released DC was further visually confirmed by Live/Dead cell staining assay. As shown in Fig. 3g, longer HIFU irradiation resulted in more cell death (red fluorescence). In comparison to DLM@PLGA NPs (Additional file 1: Figure S8), DC/DLM@PLGA NPs could induce more acute cell necrosis under HIFU irradiation which was ascribed to the down-regulated HSPs level in DC-treated 4T1 tumor cells. To verified the detailed molecular mechanisms, intracellular ATP and HSPs expression levels were then further detected by using ATP kit and Western blotting analysis, respectively. As shown in Fig. 3h, after being incubated with DC/DLM@PLGA NPs, striking decrease in intracellular ATP content was observed with increased incubation time while the ATP level of normal cells after treated with DC/DLM@PLGA NPs was slightly higher than tumor cells [40, 41]. Due to its highly ATP-dependent performance, the intracellular HSP70 synthesis was further determined by typical Western-blot assay and significant reductions of HSP70 levels were observed in 4T1 tumor cells treated with DC/DLM@PLGA NPs for 24 and 48 h, respectively (Fig. 3i). Thus, the mechanism of thermosensitive tumor cells could be attributed to the fact that the released DC molecules inhibited the glucose uptake of tumor cells and subsequently sensitized tumor cells to HIFU surgery by down-regulating the expression of ATP-dependent thermoresistant HSPs (Fig. 3j).

**Fig. 4:**
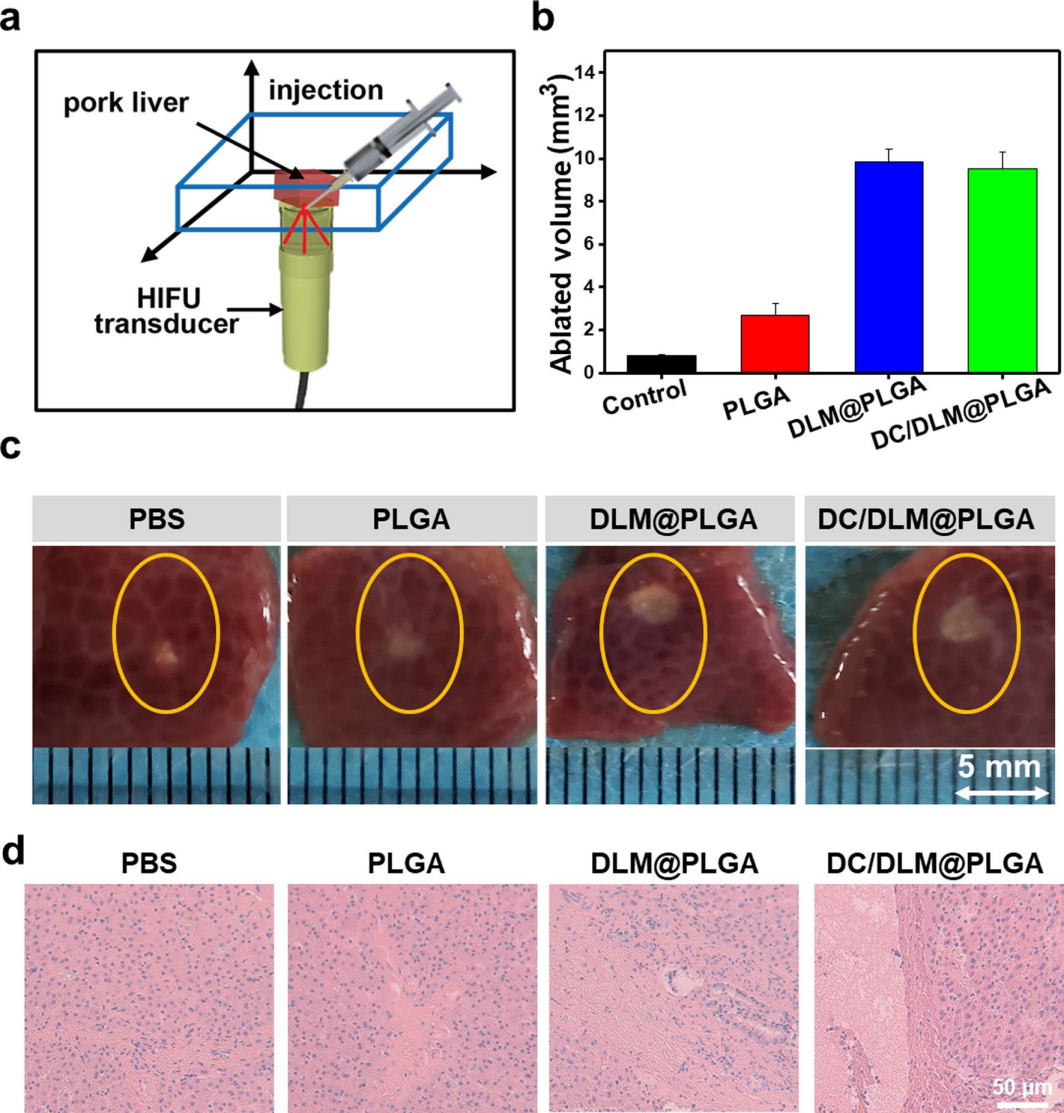
**a** Schematic diagram of experimental setup for in vitro pig liver ablation. **b** Digital photographs of the ablation areas of pig livers after injection of 200 µL solution of PBS, PLGA, DLM@PLGA, or DC/DLM@PLGA NPs and irradiation with HIFU at 25 W and 50% duty cycle for 2 min. **c** Corresponding calculation of the ablation volumes. **d** H&E histological staining of pork liver. Scale bar: 50 μm

Encouraged by the phase transition capacity of DLM after being heated, DLM-based NPs are supposed to enhance the tumor ablation through DLM vaporization induced energy deposition by both thermal and mechanical effect of HIFU irradiation. After injecting with 100 µL of PBS, PLGA NPs, DLM@PLGA NPs or DC/DLM@PLGA NPs solution, the pork livers were irradiated with HIFU (25 w, 50% duty cycle, 3 s on and 3 s off) for 2 min (Fig. 4a). The area of thermal ablation in each liver slice was then measured for characterizing the synergistic ablation effect. The digital photographs (Fig. 4b) and calculated volume of thermal ablation areas (Fig. 4c) clearly showed that the ablated areas of pork livers with DLM-encapsulated PLGA NPs (including DLM@PLGA and DC/DLM@PLGA NPs) were significantly larger than those groups without DLM, confirming the enhanced ablation effect of vaporized DLM during localized HIFU surgery. To deeper understand the cell destruction process in the HIFU irradiated zone, typical H&E histological staining of pork liver slices were then further carried out. As shown in Fig. 4d, in comparison to control groups without treated with volatile DLM molecules, the typical cytological structures (such as cell membrane, nuclei) of liver cells were destructed at the HIFU irradiated central zone in the DLM-containing groups, indicating a typical feature of coagulative necrosis [42, 43].

**Fig. 5:**
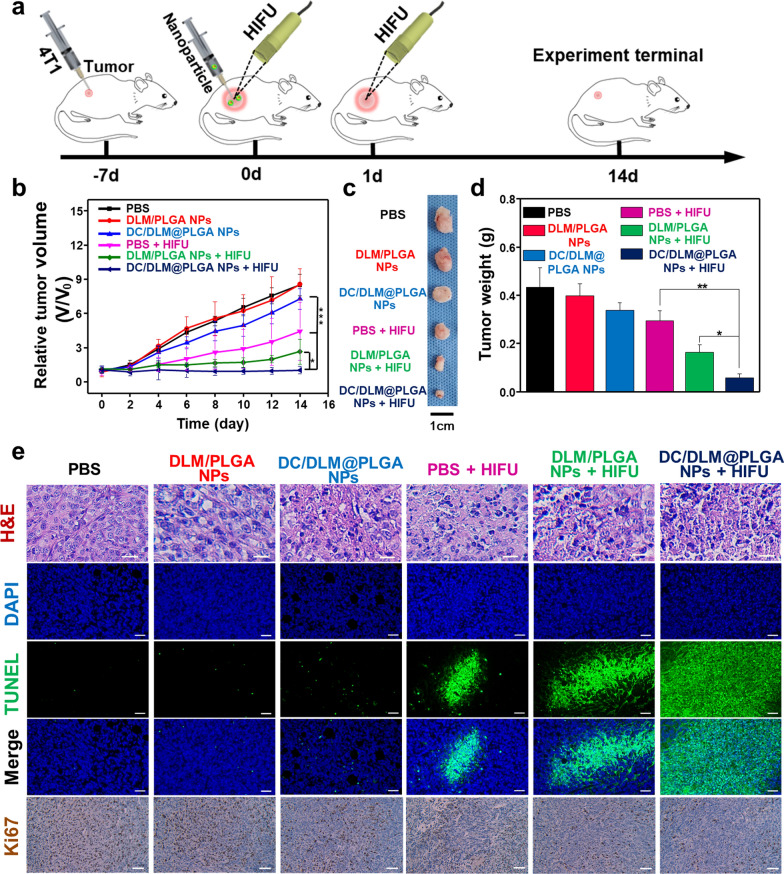
**a** Schematic illustration for experimental design. **b** Changes in tumor volumes in different groups of tumor-bearing mice after treatment (*p < 0.05, **p < 0.01). **c** Typical images to tumor tissues. **d** Average tumor weight obtained on the 14th day (*p < 0.05, **p < 0.01); (e) Typical H&E, TUNEL and Ki67 staining of tumor slices. Scale bar: 50 μm

**Fig. 6:**
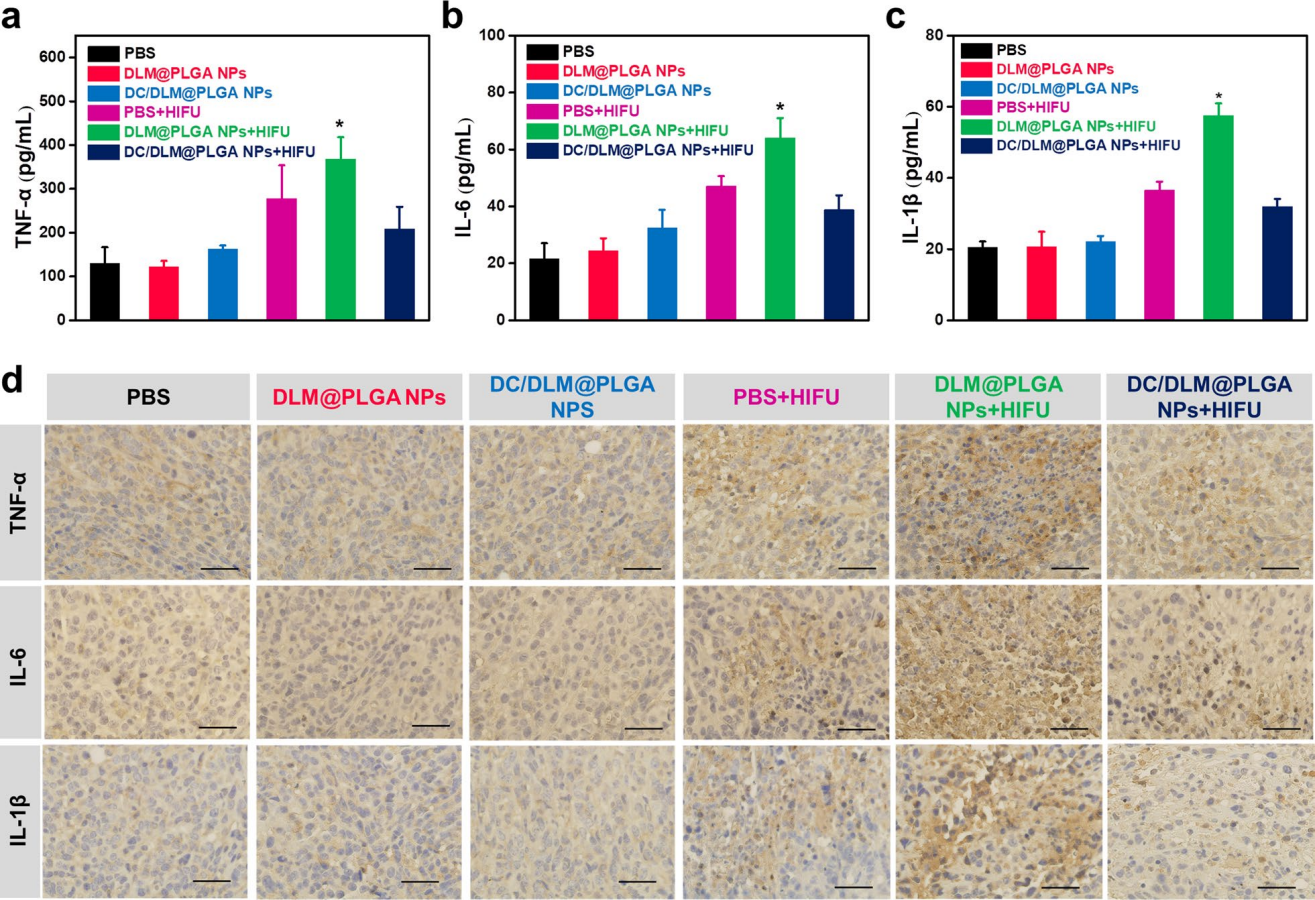
Detection of serum inflammatory cytokines, including **a** TNF-α, **b** IL-6 and **c** IL-1β in BALB/c mice after different treatments (*p < 0.05, **p < 0.01). **d** Immunohistochemical staining of typical inflammatory cytokines. Scale bar: 50 μm

In vivo tumor inhibition efficacy of HIFU surgery combined with DC/DLM@PLGA NPs was then further evaluated by 4T1 breast tumor-bearing mice. As shown in Fig. 5a, the mice were randomly divided into six groups treated with PBS, DLM/PLGA NPs, DC/DLM@PLGA NPs, PBS + HIFU, DLM/PLGA NPs + HIFU, and DC/DLM@PLGA NPs + HIFU, respectively. The treatment settings was shown in Additional file 1: Figure S9. Without HIFU irradiation, negligible tumor inhibition effect could be found in the group treated with DLM@PLGA NPs, indicating the good biocompatibility of PLGA-based nanocarriers as previously reported [44, 45]. As shown in Additional file 1: Figure S10, an in vivo imaging system (IVIS) was further used to examine the distribution of fluorescent Cy5.5@PLGA NPs (which was used as a fluorescent substitution of PLGA-based NPs in this study) in tumor-bearing mice. After intratumoral injection, the Cy5.5@PLGA NPs were almost entirely retained at the tumor sites rather than moving to other vital organs, guaranteed the negligible side effects. In consistence with the in vitro cell proliferation-inhibiting performance, the tumor growth in the DC/DLM@PLGA NPs treated group was slightly inhibited (Fig. 5b). Satisfactorily, either tumor volume or weight has been observed to be significantly decreased in the presence of HIFU irradiation (Fig. 5c and d). As verified in in vitro experiments, DLM@PLGA NPs exhibited enhanced tumor inhibition efficacy due to the DLM vaporization induced energy deposition under HIFU irradiation, which was supported by a significant increase in the grayscale of ultrasound images after HIFU exposure (Additional file 1: Figure S11), and the further cooperation with DC yielded the optimal tumor inhibition effect without obvious recurrence during the two weeks treatment period. Moreover, typical haematoxylin–eosin (H&E) staining of the tumor slices confirmed that cells appeared obviously shrunk and nuclei ruptured after DC/DLM@PLGA + HIFU treatment, which were also observed from the standard TUNEL and Ki-67 staining results (Fig. 5e). Although hyperthermia has achieved remarkable efficacy in tumor treatment, the acute inflammation induced by the release of intracellular ingredients associated with high temperature also may lead to tumor recurrence and metastasis due to the adverse inflammation. As DC is an anti-inflammatory drug widely used in clinic practice by inhibiting cyclooxygenase synthesis [46, 47], in the mean time, numerous studies have reported that in an inflammatory environment, massively expressed neutrophil extracellular bactericidal networks (NETs) promote tumor metastasis by inducing inflammatory responses characterized by up-regulation of COX2 (cyclooxygenase 2) [48]. Thus, DC-encapsulated DLM@PLGA NPs with the property of inhibition to inflammation after HIFU hyperthermia was desired, which may contribute to the suppression of tumor recurrence. After being irradiated by HIFU for 24 h, the serum of mice was taken to detect its levels of inflammatory cytokines. As shown in Fig. 6a–c, HIFU-treated mice with injection of PBS or DLM@PLGA NPs had been detected with significant increases in the level of TNF-α, IL-6, and IL-1β. In dramatic contrast, benefiting from the anti-inflammatory property of DC, the levels of inflammatory cytokines in the DC/DLM@PLGA NPs + HIFU group was significantly decreased. Similarly, immunohistochemical staining of TNF-α, IL-6, and IL-1β also confirmed that DC/DLM@PLGA NPs could effectively alleviate the inflammation caused by HIFU hyperthermia (Fig. 6d). Moreover, the results of body weight, pathological examination and blood biochemical analysis of the main organs of mice after the 14 days treatments showed that DC/DLM@PLGA NPs were negligible toxic which could have great potential for clinical applications (Additional file 1: Figures S12–S14). Furthermore, survival analysis also illustrated better therapeutic activity of HIFU jointed with DC/DLM@PLGA NPs (Additional file 1: Figure S15). After counting the changes in tumor volume of each mouse after treatment in different groups, we found that compared with other groups, the tumor recurrence of the mice in the DC/DLM@PLGA NPs group was delayed to a certain extent, which may be attributed to the anti-inflammatory effect of DC (Additional file 1: Figure S16).

## Conclusions

In summary, dual-functionalized DC with anti-inflammation and glycolysis-inhibition abilities were successfully co-encapsulated with phase-change medium DLM in PLGA NPs to realize improved HIFU surgery without causing adverse inflammation. The solid-liquid-gas transition of DLM would not only enhance the energy deposition in tumor region during HIFU surgery but also promote the release of encapsulated DC. As a welfare, the released DC molecules inhibited the glucose uptake of tumor cells and subsequently sensitized tumor cells to HIFU surgery through down-regulating the expression of thermo-resistant HSPs. Meanwhile, the anti-inflammatory DC could effectively reduce the occurrence of adverse inflammation caused by HIFU induced coagulative necrosis. Thus, as a proof-of-concept study, our work provides a efficient strategy for simultaneously improving the curative efficiency and diminishing the harmful inflammatory responses of clinical HIFU surgery.

## Supplementary Information

**Additional file 1:** **Figure S1.** The UV-Vis absorption spectrum and standard curve of DC. **Figure S2.** Photographs of DC/DLM@PLGA nanoparticles dispersed in de-ionized water (DI water), PBS solution (pH 6.0, 6.5, 7.4) and DMEM culture medium. **Figure S3.** Photographs of the device used to monitor the temperature changes of different dispersions (PLGA, DLM@PLGA, DC/DLM@PLGA) under HIFU irradiation. **Figure S4.** The temperature variation profiles of PBS solution under different HIFU power. **Figure S5.** In vitro thermal infrared images of PBS solution containing PLGA, DLM@PLGA or DC/DLM@PLGA after different HIFU irradiation times. **Figure S6.** Photographs of a rubber tube containing PBS solution and DC/DLM@PLGA dispersion after 30 min in a 60 °C water bath. **Figure S7.** Cytotoxicity of free DC with different concentrations to HUVECs and 4T1 cells. **Figure S8.** Live/Dead staining imaging of 4T1 cells treated with PBS, DLM@PLGA, DLM@PLGA + HIFU (60 s) and DLM@PLGA + HIFU (120 s). **Figure S9.** Photograph of the process of anti-tumor treatment of 4T1 tumor-bearing mice with DC/DLM@PLGA + HIFU irradiation. **Figure S10.** Ex vivo organ images of tumor bearing-mice injected with fluorescent Cy5.5 @PLGA NPs monitored at 0 h, 1 h, 4 h, 8 and 24 h. **Figure S11.** Typical B-mode ultrasound images of tumors which were injected with PBS or DC/DLM@PLGA NPs under various treatments. **Figure S12.** Body weight change of 4T1 tumor-bearing mice after various treatments in 14 days. **Figure S13.** H&E images of major organs extracted from different groups after treatments as indicated. Scale bar: 50 μm. **Figure S14.** Blood routine and biochemical indexes of mice treated with different treatments. **Figure S15.** Survival rate of the 4T1 tumor bearing-mice with various treatments as indicated (n = 5). **Figure S16.** Changes in tumor volume of 4T1 tumor bearing-mice of various treatments as indicated (n = 5).

## Data Availability

All data generated or analyzed during this study are included in this article.

## Abbreviations

SEM: Scanning electron microscopy
TEM: Transmission electron microscopy
MTT: Methyl thiazolyl tetrazolium
CCK-8: Cell counting kit-8
PVA: Hydrophilic polyvinyl alcohol
