# Supplementary material for: Three birds with one stone: co-encapsulation of diclofenac and DL-menthol for realizing enhanced energy deposition, glycolysis inhibition and anti-inflammation in HIFU surgery
Source: J Nanobiotechnology. 2022 May 6;20:215. doi: 10.1186/s12951-022-01437-2 (PMC9074192; doi:10.1186/s12951-022-01437-2)
Supplement: Supplementary file 1 — Additional file 1: Figure S1. The UV-Vis absorption spectrum and standard curve of DC. Figure S2. Photographs of DC/DLM@PLGA nanoparticles dispersed in de-ionized water (DI water), PBS solution (pH 6.0, 6.5, 7.4) and DMEM culture medium. Figure S3. Photographs of the device used to monitor the temperature changes of different dispersions (PLGA, DLM@PLGA, DC/DLM@PLGA) under HIFU irradiation. Figure S4. The temperature variation profiles of PBS solution under different HIFU power. Figure S5. In vitro thermal infrared images of PBS solution containing PLGA, DLM@PLGA or DC/DLM@PLGA after different HIFU irradiation times. Figure S6. Photographs of a rubber tube containing PBS solution and DC/DLM@PLGA dispersion after 30 min in a 60 °C water bath. Figure S7. Cytotoxicity of free DC with different concentrations to HUVECs and 4T1 cells. Figure S8. Live/Dead staining imaging of 4T1 cells treated with PBS, DLM@PLGA, DLM@PLGA + HIFU (60 s) and DLM@PLGA + HIFU (120 s). Figure S9. Photograph of the process of anti-tumor treatment of 4T1 tumor-bearing mice with DC/DLM@PLGA + HIFU irradiation. Figure S10. Ex vivo organ images of tumor bearing-mice injected with fluorescent Cy5.5 @PLGA NPs monitored at 0 h, 1 h, 4 h, 8 and 24 h. Figure S11. Typical B-mode ultrasound images of tumors which were injected with PBS or DC/DLM@PLGA NPs under various treatments. Figure S12. Body weight change of 4T1 tumor-bearing mice after various treatments in 14 days. Figure S13. H&E images of major organs extracted from different groups after treatments as indicated. Scale bar: 50 μm. Figure S14. Blood routine and biochemical indexes of mice treated with different treatments. Figure S15. Survival rate of the 4T1 tumor bearing-mice with various treatments as indicated (n = 5). Figure S16. Changes in tumor volume of 4T1 tumor bearing-mice of various treatments as indicated (n = 5). [file 12951_2022_1437_MOESM1_ESM.docx]

Additional file 1

**Three Birds with One Stone: Co-Encapsulation of Diclofenac and DL-Menthol for Realizing Enhanced Energy Deposition, Glycolysis Inhibition and Anti-Inflammation in HIFU Surgery**

Haitao Wu^1, †^, Hu Zhou^2, †^, Wenjie Zhang^1^, Ping Jin^2,^ *, Qianqian Shi^1^, Zhaohua Miao^1^, Hua Wang^3,^ * and Zhengbao Zha^1,^ *

*^1^* School of Food and Biological Engineering, School of Instrument Science and Opto-Electronics Engineering, Hefei University of Technology, Hefei, Anhui 230009, P. R. China.

*Correspondence: [pingjin68@smu.edu.cn](mailto:pingjin68@smu.edu.cn); [wanghua@ahmu.edu.cn](mailto:wanghua@ahmu.edu.cn); [zbzha@hfut.edu.cn](mailto:zbzha@hfut.edu.cn).

^†^Haitao Wu and Zhou Hu contributed equally.

**Experimental Section**

**Materials and reagents**

PLGA was gifted from Hefei Zhongren Technology Co., Ltd. Diclofenac (DC) was purchased from Tokyo Chemical Industry (TCI). Polyvinyl alcohol (PVA) was purchased from Alfa Aesar. _DL_-Menthol (DLM), methylthiazolyldiphenyl-tetrazolium bromide (MTT), calcelin-AM and propidium iodide (PI) were supplied by Sigma-Aldrich. All organic reagents, including ethanol, DMSO and CH_2_Cl_2_, were purchased from Sinopharm Chemical Reagent Company. DMEM and RPMI 1640 cell culture medium were both obtained from HyClone Company. Deionized (DI) water (18.2 MΩ/cm) used in the experiment was prepared by using a Milli-Q Gradient System.

**Synthesis of DC/DLM@PLGA NPs**

DC/DLM@PLGA NPs were synthesized by the typical oil-in-water emulsion method. Specifically, 0.8 mL DMSO with 20 mg of DC was firstly added to 4.0 mL CH_2_Cl_2_, in which 100 mg of PLGA and 50 mg of DLM were dissolved, and then the mixed solution was added into 20 mL PVA solution (2.0 wt%). Subsequently, under continuous probe sonication (5 min), the above-mentioned immiscible mixtures formed a stable oil-in-water emulsion. Finally, the residual CH_2_Cl_2_ was evaporated by magnetic stirring at room temperature for 4 h. The preparation process of DLM@PLGA or PLGA NPs was basically the same as that of DC/DLM@PLGA NPs; the only difference is that DC or DLM was not added during the process of dissolving PLGA with CH_2_Cl_2_.

***In vitro* DC release performance from DC/DLM@PLGA NPs**

20 mg of DC/DLM@PLGA NPs was dispersed in 2.0 mL of PBS solution and then packaged into a dialysis bag (Mw = 3000), which was immersed into a beaker containing 498 mL of PBS under magnetic stirring. PBS (2.0 mL) at predetermined time intervals was extracted from the beaker to measure UV−vis absorption. In addition, 2 mL of fresh PBS solution was added to the beaker to maintain a constant release volume. To investigate the influence of HIFU irradiation on DC release, the dialysis bag was irradiated for 5 min with HIFU (25 W, duty cycle: 50%, HIFU ON 3S/OFF 3S), with the focus of HIFU (focal length: 16 mm) located in the dialysis bag containing the DC/DLM@PLGA. Under the same conditions, DLM@PLGA nanoparticles loaded with DC without HIFU irradiation served as a control.

***In vitro* ultrasound imaging**

The synthesized DC/DLM@PLGA NPs (10 mg/mL) were packed into a rubber tube 0.6 cm in diameter and immersed in a beaker containing 1.5 L of degassed water. Then, an ultrasonic diagnostic detector (frequency: 6.6 MHz; frame frequency: 33 Hz) was fixed above the rubber tube to capture ultrasound images of DC/DLM@PLGA NPs at different time intervals (1, 5, 10, 20, and 30 min). The total apparatus was placed on a heating stirrer, which was set to a constant temperature of 60 °C. Degassed PBS buffer solution was used as a control.

***In vitro* cytotoxicity assays**

HUVECs were used here to evaluate the cytotoxicity of DC/DLM@PLGA NPs. Cells were seeded in 96-well plates at a density of 5×10^3^ cells per well. After culturing for 24 h, the medium was replaced with fresh DMEM cell culture medium containing DC/DLM@PLGA NPs with gradient concentrations. The cytotoxicity of the as-prepared NPs was then characterized by standard MTT or CCK-8 assays.

4T1 breast cancer cells were used to evaluate the cell killing effect with/without HIFU irradiation. Cells with gradient concentrations of DC/DLM@PLGA NPs were irradiated by HIFU (power: 25 W, irradiation time: 5 min, duty cycle: 50%), and another 2 min of HIFU irradiation was enforced. After incubating for additional 24 h, the cell viabilities were then characterized through standard MTT and Live/Dead cell staining assays.

**Determination of intracellular glucose and ATP levels**

To investigate the inhibitory effect of DC on the glucose metabolism of tumor cells, 4T1 cells and HUVECs (used here as a control) were seeded in 6-well plates at a density of 5 × 10^5^ cells per well. After culturing for 24 h or 48 h, the cells were treated with DC/DLM@PLGA NPs with/without 5 min HIFU irradiation. Subsequently, the cells were lysed by ultrasound (200 W, 30 min) and boiled in a boiling water bath for 10 minutes. After collecting the supernatant by centrifugation, the intracellular glucose level of the cells was detected using a glucose content assay kit (BC2500, Solarbio).

For the determination of intracellular ATP levels, cells treated with various groups were lysed by cell lysis buffer. After collecting the supernatant by centrifugation (12000 rpm, 4 °C), the intracellular ATP levels of the cells were detected using an ATP assay kit (S0026, Beyotime).

**Determination of HSP70 levels**

Briefly, 4T1 cells were seeded in 6-well plates at a density of 5 × 10^5^ cells per well for 24 h, followed by incubating with DC/DLM@PLGA NPs or PBS for different time periods (0 h, 24 h, 48 h). The cells were then lysed and centrifuged to collect the supernatant. After the protein concentration in collected supernatant was determined by typical BCA method, loading buffer was added to the supernatant and boiled for 10 minutes. After being incubated with the primary antibody rabbit anti-human HSP70 overnight at 4 °C, the PVDF membranes were washed with 1× TBST and incubated with the secondary antibody for 1 h. Speciﬁc proteins were detected by using enhanced chemiluminescence. GAPDH was typically employed as a protein loading control.

***In vitro* evaluation of the HIFU ablation effect on pork livers**

A portable high-intensity focused ultrasound ablation device (gifted from HaiYing Medical Technology Co., Ltd, Wuxi, China) was employed for in vitro ablation of pork livers. The experimental steps were as follows. First, the HIFU transducer with a focal length of 16 mm, which was weared with a plastic sleeve with a diameter of 21 mm, was filled with degassed water. Subsequently, 0.2 mL of PBS solution, PLGA NPs, DLM@PLGA NPs and DC/DLM@PLGA NPs in PBS (all 10 mg/mL) were injected into a plastic sleeve, and then degassed pork livers were placed onto the plastic sleeve. After exposure to HIFU for 3 min (25 W), pork livers were removed, and the extent of the coagulative necrosis area was measured. The necrotic volumes were calculated using the following equation (1):

$V=\pi\times L\times W^{2}/6$· (1)

where V is the necrotic volume and L and W are the maximum length of necrotic tissues and maximum width of necrotic tissues, respectively.

To verify the occurrence of coagulation necrosis after HIFU ablation, HIFU-irradiated pork liver sections was collected and stained with haematoxylin and eosin (H&E).

***In vivo* biodistribution study**

To investigate the biodistribution performance of NPs, PLGA NPs was labeled with Cy5.5 molecules, a commonly used near-infrared fluorescent dye. 4T1 tumor bearing-mice were intratumorally injected with as-prepared Cy5.5@PLGA NPs (100 μL per mouse). Subsequently, the mice were euthanized at the predetermined time points (0, 1, 4, 8 and 24 h) and then the tumors and fatal organs, including heart, liver, spleen, lung and kidney, of the mice were dissected, isolated and further imaged by an *in vivo* imaging system (IVIS, Perkin Elmer, excitation wavelength was set at 675 nm, emission wavelength was 730 nm).

***In vivo* ultrasound imaging**

To further evaluate the enhanced ultrasonic imaging effect of DC/DLM@PLGA NPs *in vivo*, 4T1 tumor-bearing mice were firstly injected intraperitoneally with chloral hydrate (4%). Then, the skin and hair in the tumor site of anesthetized mice were treated with hair removal cream and gently wiped with 75% alcohol to eliminate microbubbles. Then, the tumor sites were immersed in a degassed water sac at 37 °C of clinical HIFU device (JC-200). Adjusting the spatial location of the ultrasonic probe, the ultrasound images were taken before and after injecting 100 μL PBS solution or DC/DLM@PLGA NPs (10 mg/mL) dispersion into the tumor site of mice. After setting the irradiation power of HIFU (120 W, 2 s), the ultrasound images were captured.

***In vivo* noninﬂammatory HIFU ablation**

All animal studies were in accordance with the animal protocol approved by the Ethics Committees of the Hefei University of Technology (No. HFUT20191015001), and all procedures were in accordance with the Guidelines of the Animal Care and Use Committee of Hefei University of Technology. Female BALB/c mice (4-6 weeks) were purchased from Jinan PengYue Laboratory Animal Breeding Co. Ltd.

To further evaluate the antitumor effect *in vivo*, forty-two 4T1 tumor-bearing nude mice were randomly divided into six groups: PBS, DLM/PLGA NPs, DC/DLM@PLGA NPs, PBS + HIFU, DLM/PLGA NPs + HIFU, and DC/DLM@PLGA NPs + HIFU. After intratumoral injection with 0.2 mL of samples into the 4T1 tumors of mice (∼30 mm^3^), the tumor tissue and the probe were filled with a coupling agent to reduce sound attenuation. Finally, tumor tissue was subjected to HIFU irradiation (power: 25 W, focal length: 16 mm, duty cycle: 50%) for 5 min. The relative tumor volume and body weight of the treated mice were recorded every other day. Tumor size was measured by a caliper, and tumor volume was calculated using the following formula (2):

$V=L\times W^{2}/2$· (2)

where V is the tumor volume and L and W are the tumor length and tumor width, respectively. The relative tumor volume was calculated as 100%×V/V_1_ (where V_1_ is the tumor volume before therapy). The tumor-bearing mice were weighed, and the tumor volume was measured at specific time intervals. Simultaneously, tumor tissues, major organs (heart, liver, spleen, lung, and kidney) and serum after HIFU irradiation were collected and used. H&E staining, immunoﬂuorescence staining and Ki67 staining were used to evaluate the antitumor mechanism. Moreover, after different treatments, the serum and tumor tissue of the mice were harvested for evaluation of the levels of inflammatory factors (TNF-α, IL-6, IL-1β) in mice with an ELISA kit (Wuhan Colorful-Gene Biological Technology, Wuhan, China) and immunohistochemistry staining to confirm the anti-inflammatory effect of the prepared sample.

For biocompatibility study, female BALB/c mice (aged 4-6 weeks) were randomly divided into two groups: PBS and DC/DLM@PLGA NPs. The mice were sacrificed, and blood was taken on the 7^th^ and 21^st^ days after intravenous injection of 0.2 mL of PBS or DC/DLM@PLGA NPs to perform routine blood biochemistry measurements.

**Characterization**

The morphologies of the as-prepared DC/DLM@PLGA NPs were observed by scanning electron microscopy (SEM, Hitachi SU8020, Japan) and transmission electron microscopy (JEM-1400ﬂash, 120 kV, Japan). The hydrodynamic diameter distribution and zeta potential of DC/DLM@PLGA NPs were acquired by a Malvern Zetasizer Nano ZS 90 instrument (UK). Thermogravimetric analysis (TGA) was performed with a thermal analyzer (STA449F5 Jupiter, NETZSCH) at a heating rate of 10 °C/min under nitrogen ﬂow. The optical properties of diﬀerent samples were collected using a spectrophotometer (U-5100, Hitachi). The temperature monitoring of different samples was recorded with an infrared thermal imager (Testo 865, Testo, Germany).

**Additional data**

**
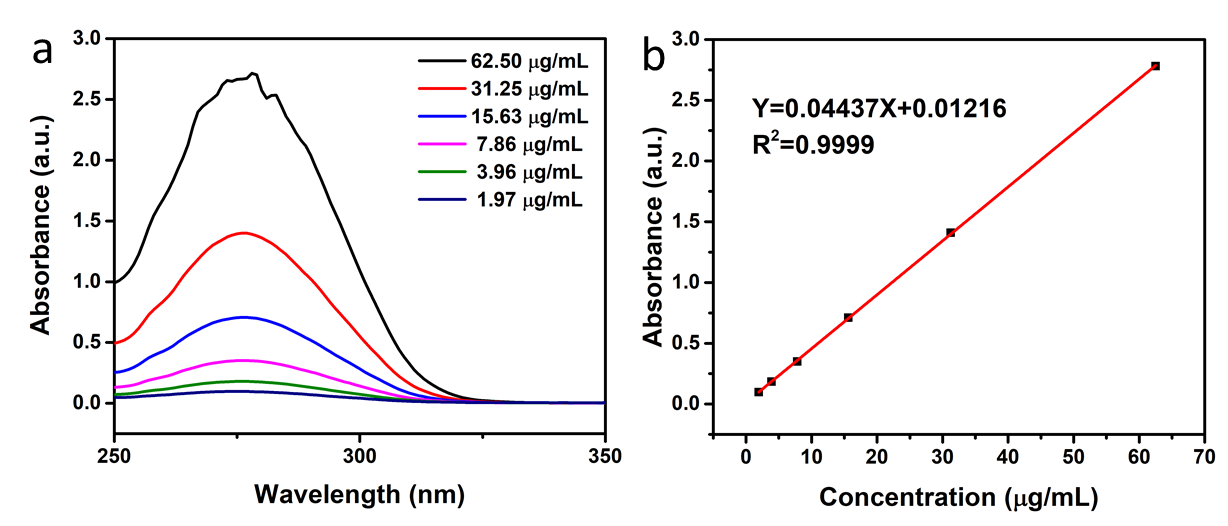
**

**Fig. S1.** The UV-Vis absorption spectra (a) and corresponded standard curve (b) of DC.


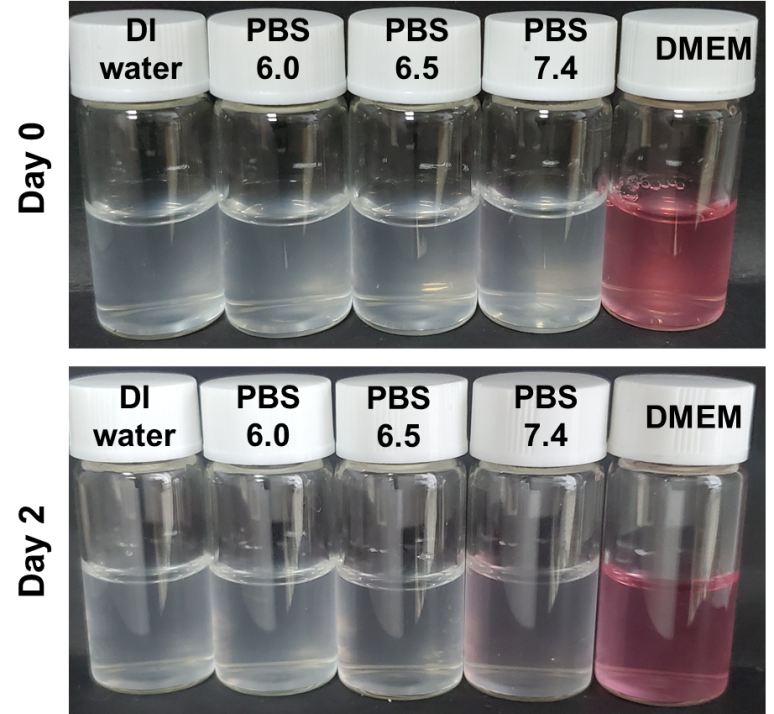


**Fig. S2.** Photographs of DC/DLM@PLGA NPs dispersed in DI water, PBS solution (pH 6.0, 6.5, 7.4) and DMEM culture medium.


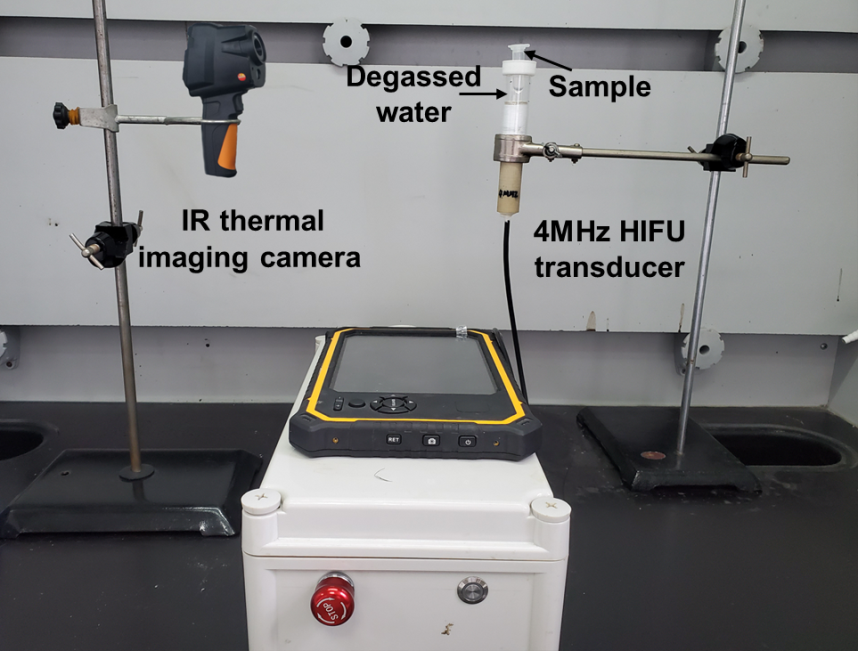


**Fig. S3.** Photograph of the device used to monitor the temperature changes of different dispersions (PLGA NPs, DLM@PLGA NPs, DC/DLM@PLGA NPs) under HIFU irradiation.

**
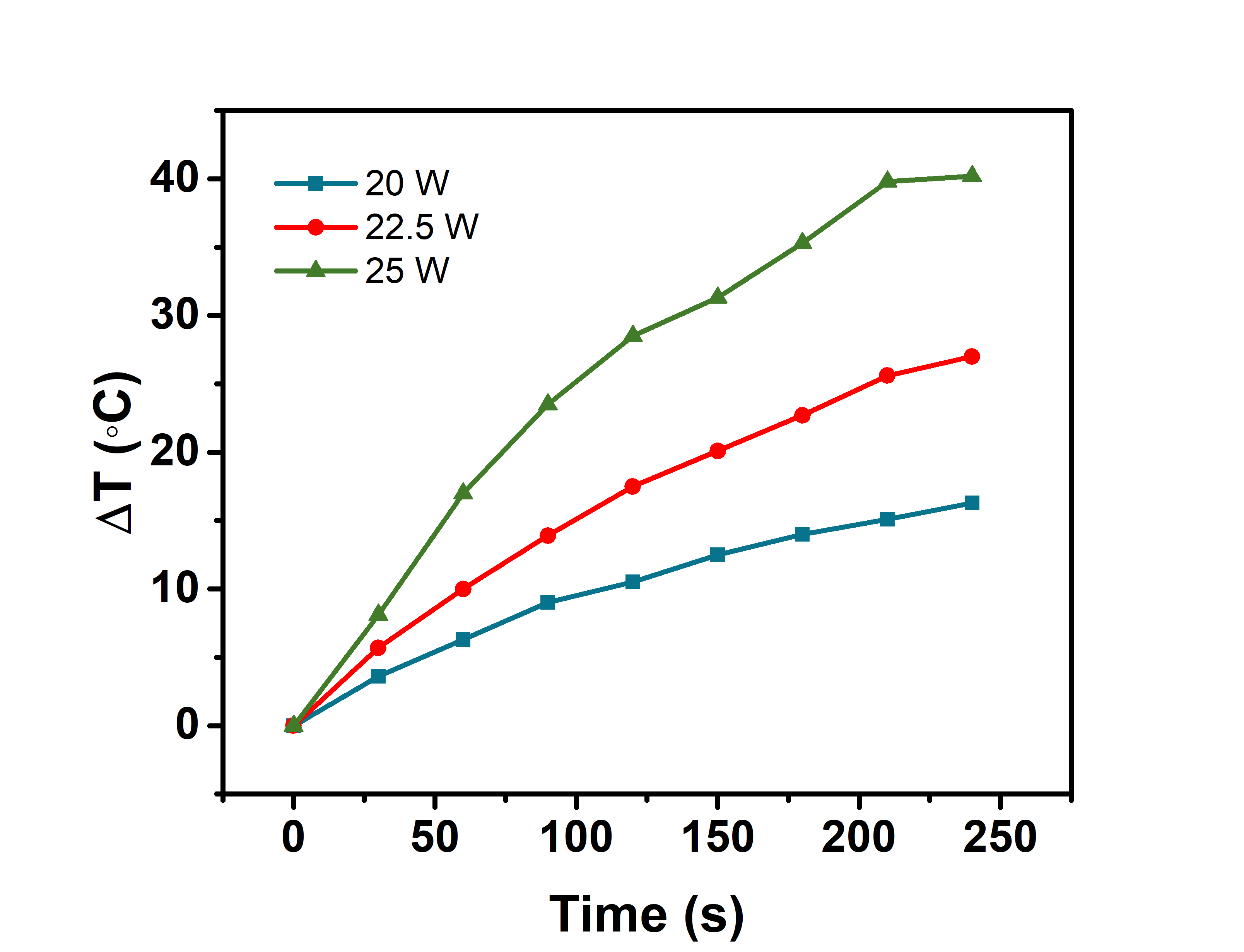
**

**Fig. S4.** The temperature variation profiles of PBS solution under irradiation with different HIFU power.


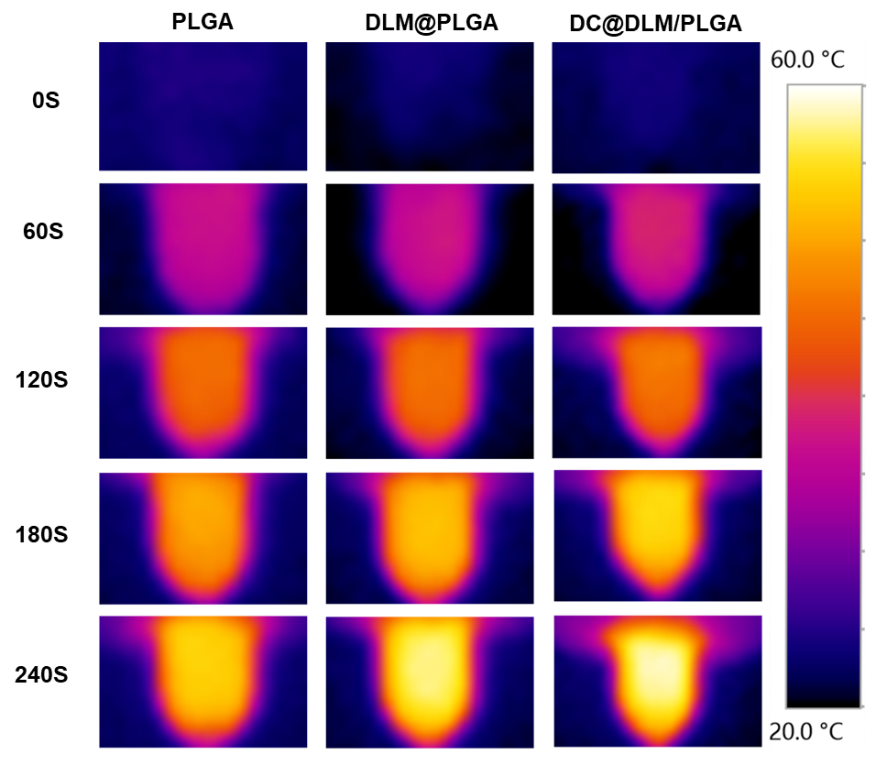


**Fig. S5.** *In vitro* thermal infrared images of PBS solution containing PLGA NPs, DLM@PLGA NPs or DC/DLM@PLGA NPs with different HIFU irradiation treatments.

**PBS**


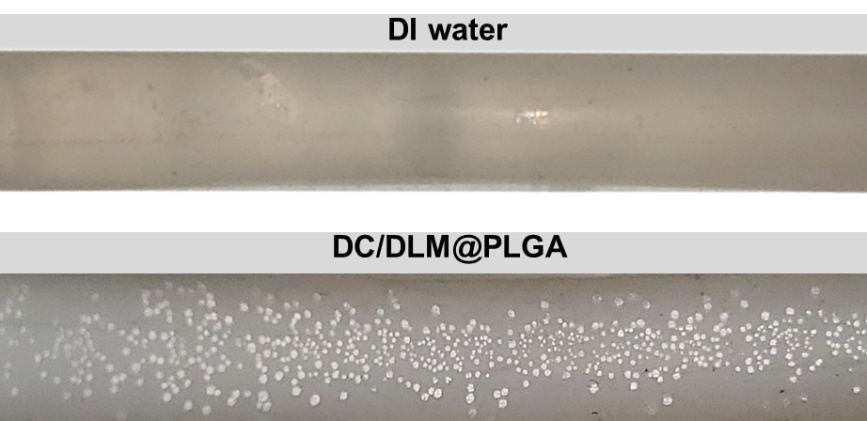


**DC/DLM@PLGA**


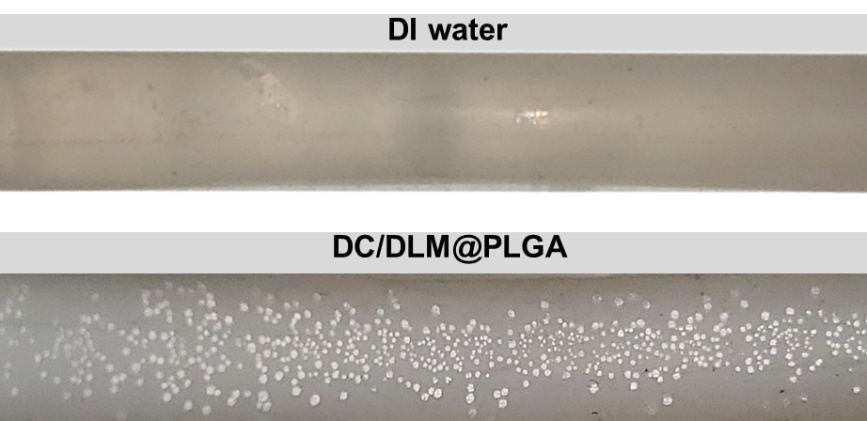


**1 cm**

**Fig. S6.** Photographs of a rubber tube containing PBS solution and DC/DLM@PLGA NPs dispersion in a 60 °C water bath for 30 min.


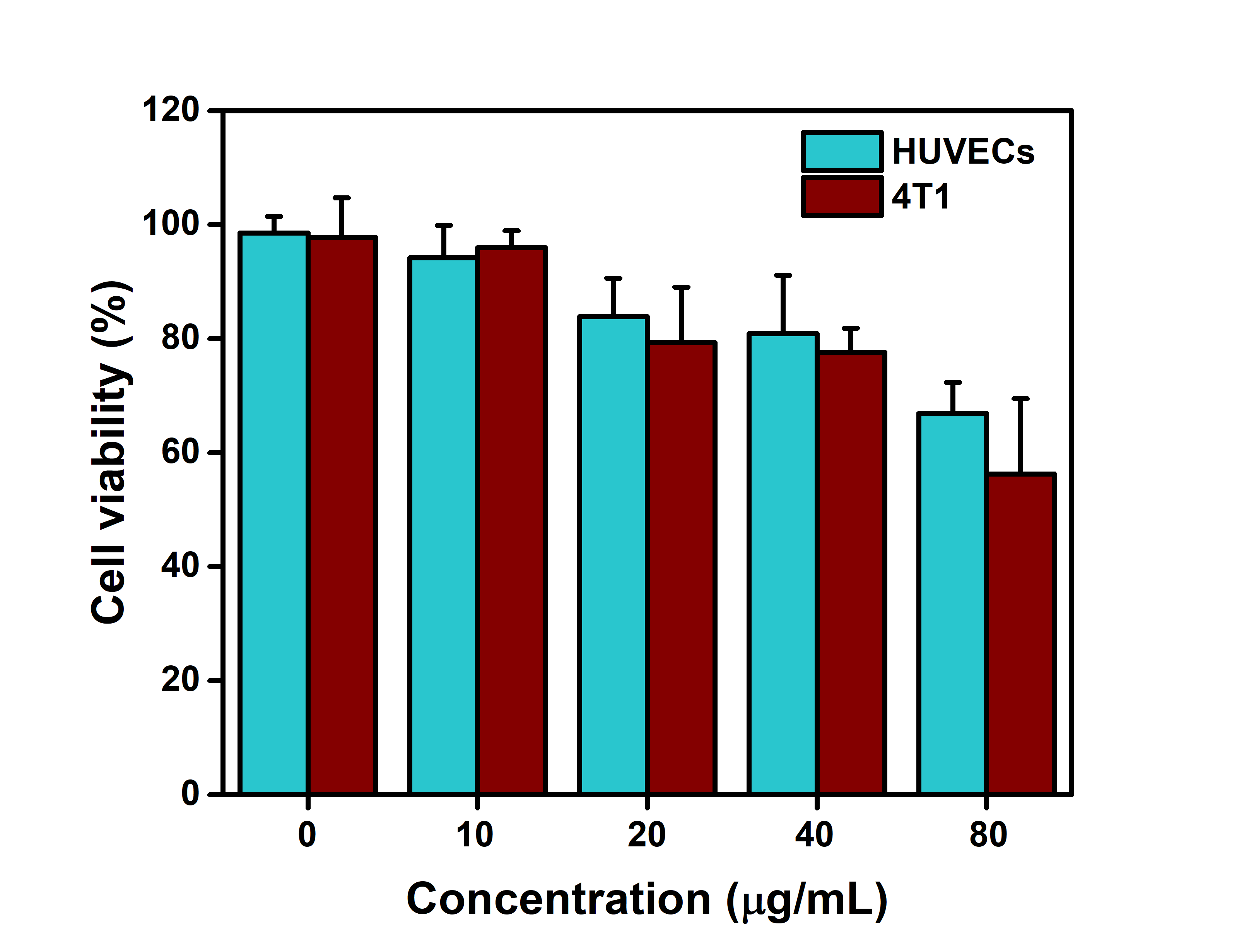


**Fig. S7.** Cytotoxicity of free DC with different concentrations to HUVECs and 4T1 cells.


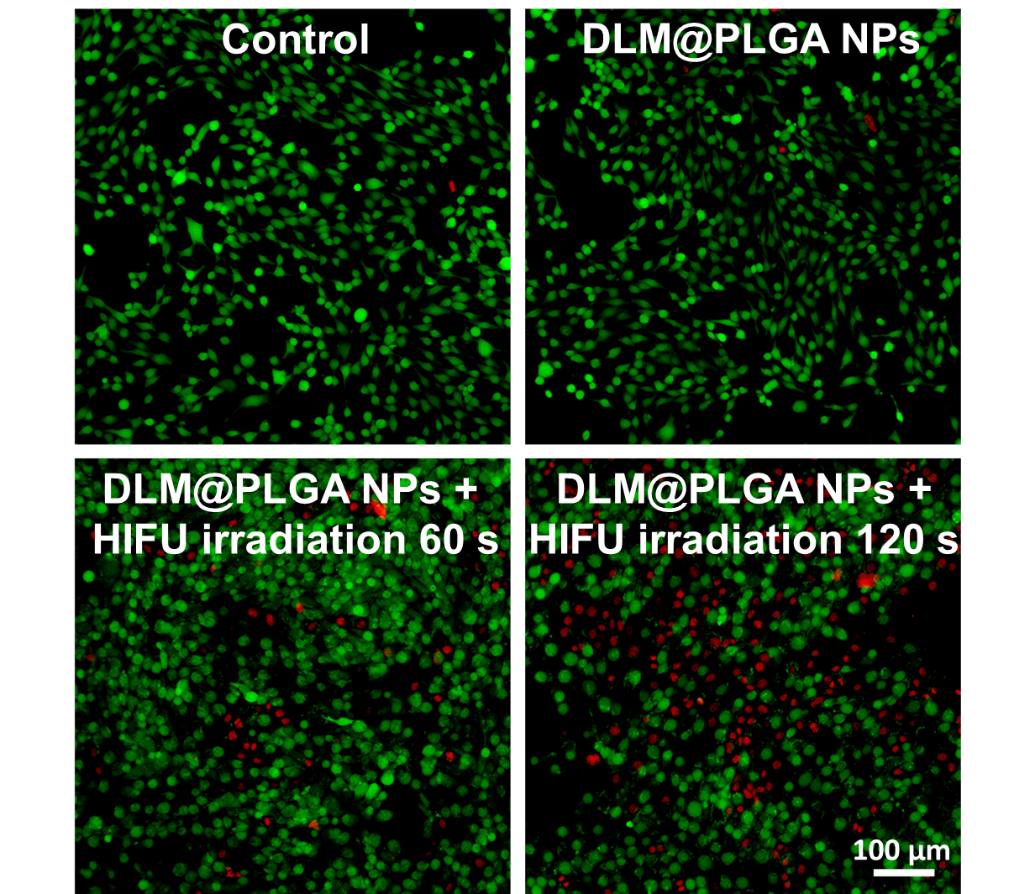


**Fig. S8.** Live/Dead staining imaging of 4T1 cells treated with PBS, DLM@PLGA NPs, DLM@PLGA NPs+HIFU (60 s) and DLM@PLGA NPs+HIFU (120 s).


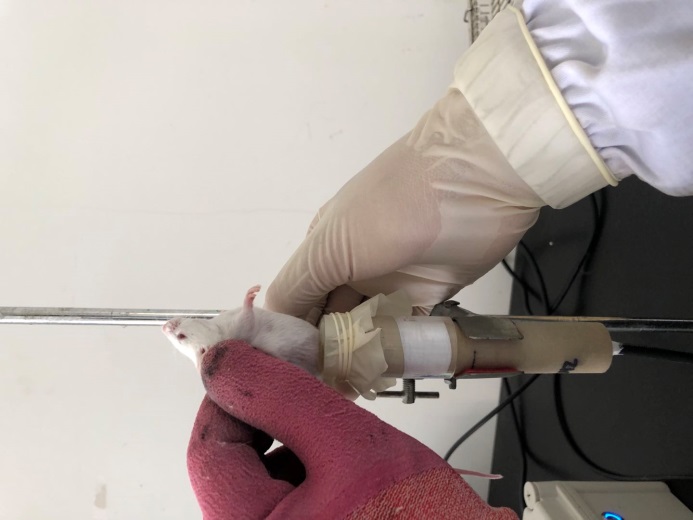


**Fig. S9.** Photograph of the process of anti-tumor treatment of 4T1 tumor-bearing mice with DC/DLM@PLGA NPs+HIFU irradiation.


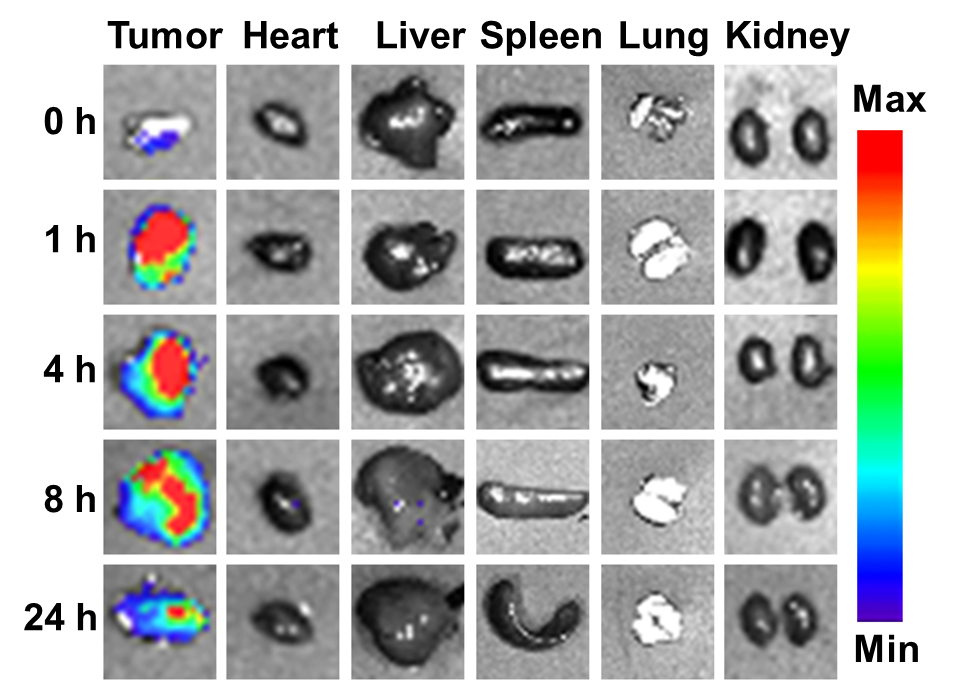


**Fig. S10.** *Ex vivo* organ images of tumor bearing-mice injected with fluorescent Cy5.5 @PLGA NPs monitored at 0h, 1h, 4 h, 8 h and 24h.


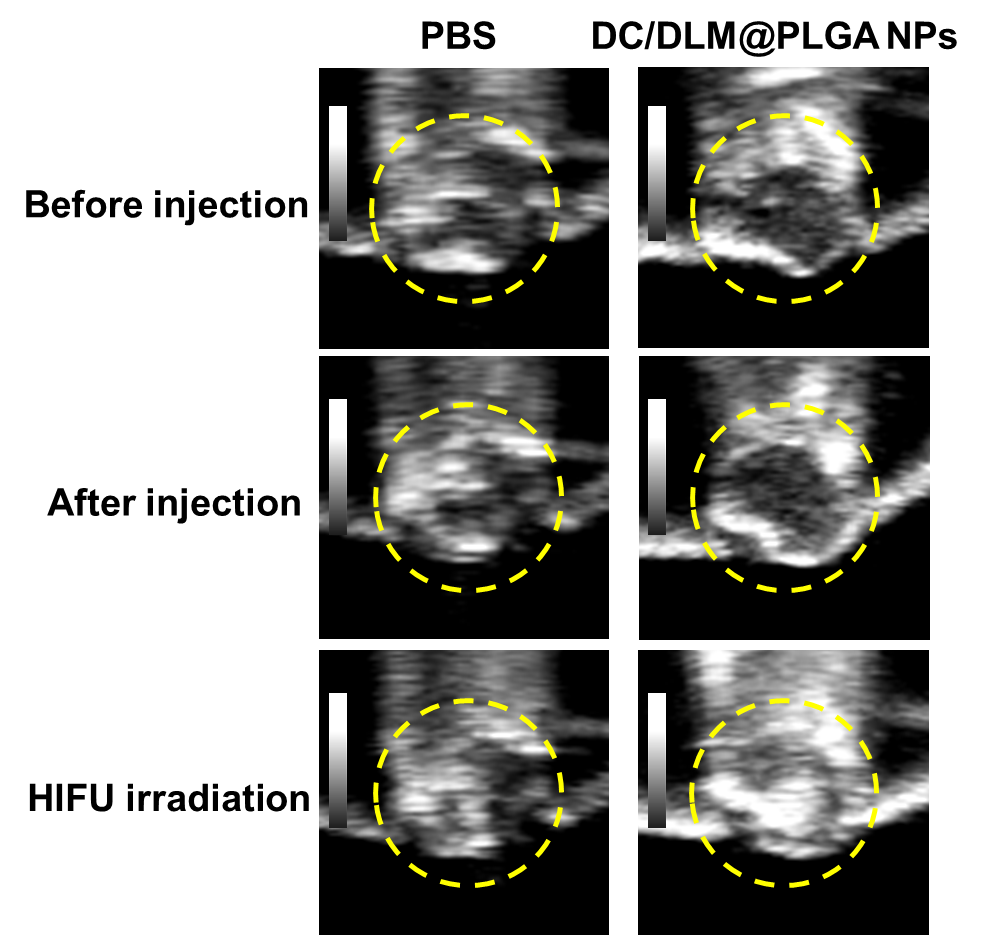


**Fig. S11.** Typical B-mode ultrasound images of tumors which were injected with PBS or DC/DLM@PLGA NPs under various treatments.


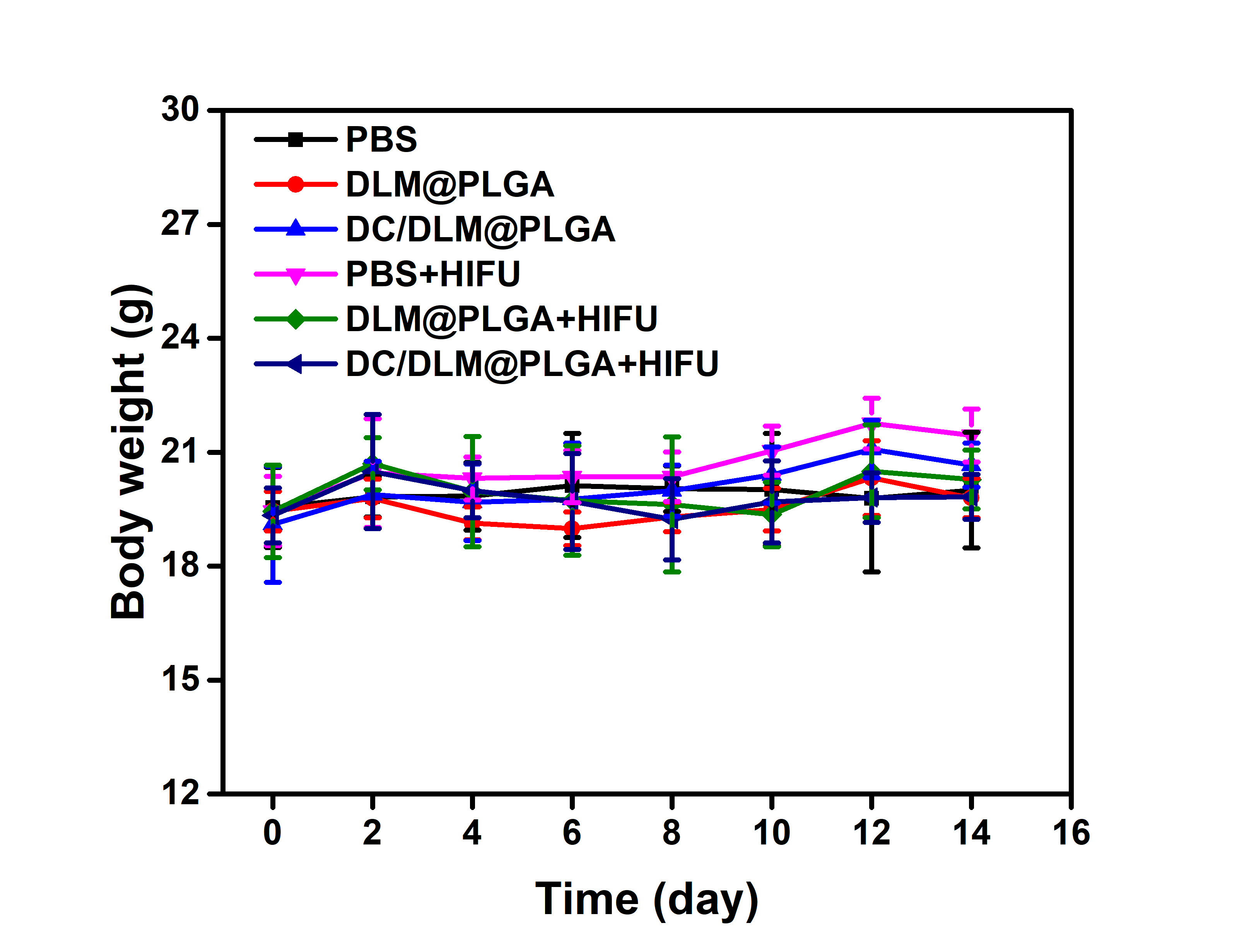


**Fig. S12.** Body weight change of 4T1 tumor-bearing mice after various treatments in 14 days.

**
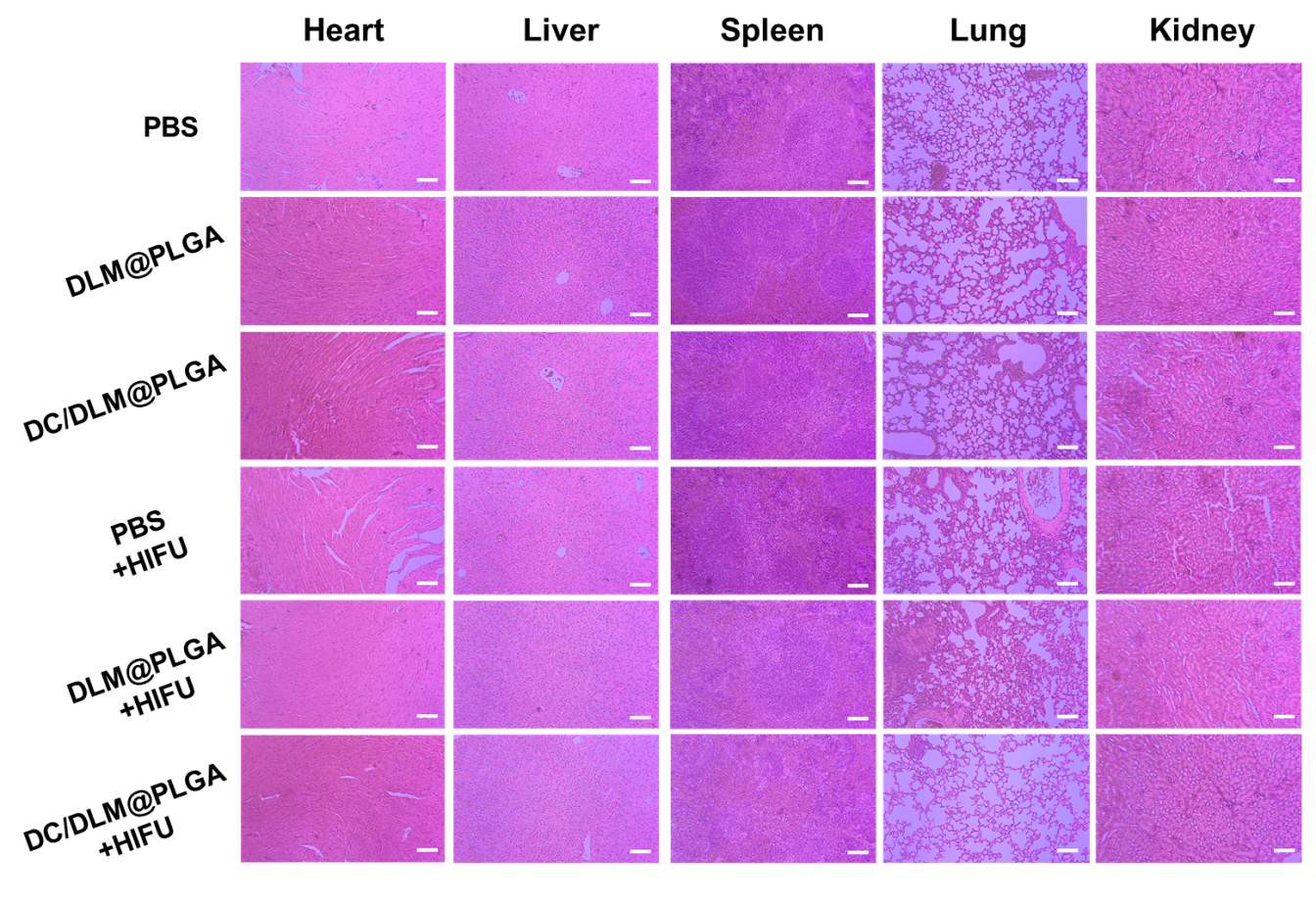
**

**Fig. S13.** H&E images of major organs extracted from different groups after treatments as indicated. Scale bar: 50 μm.


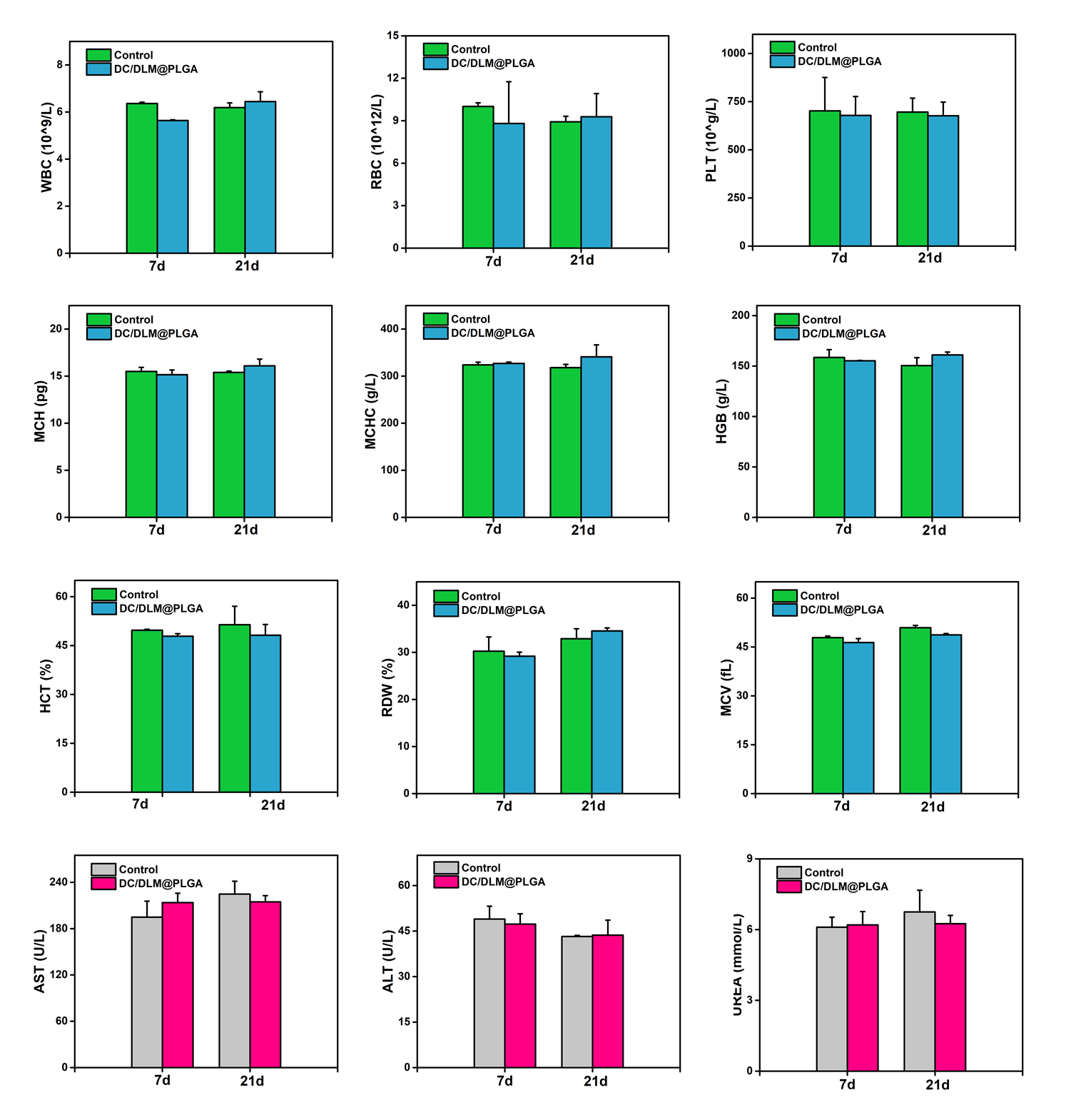


**Fig. S14.** Blood routine and biochemical indexes of mice treated with different treatments.


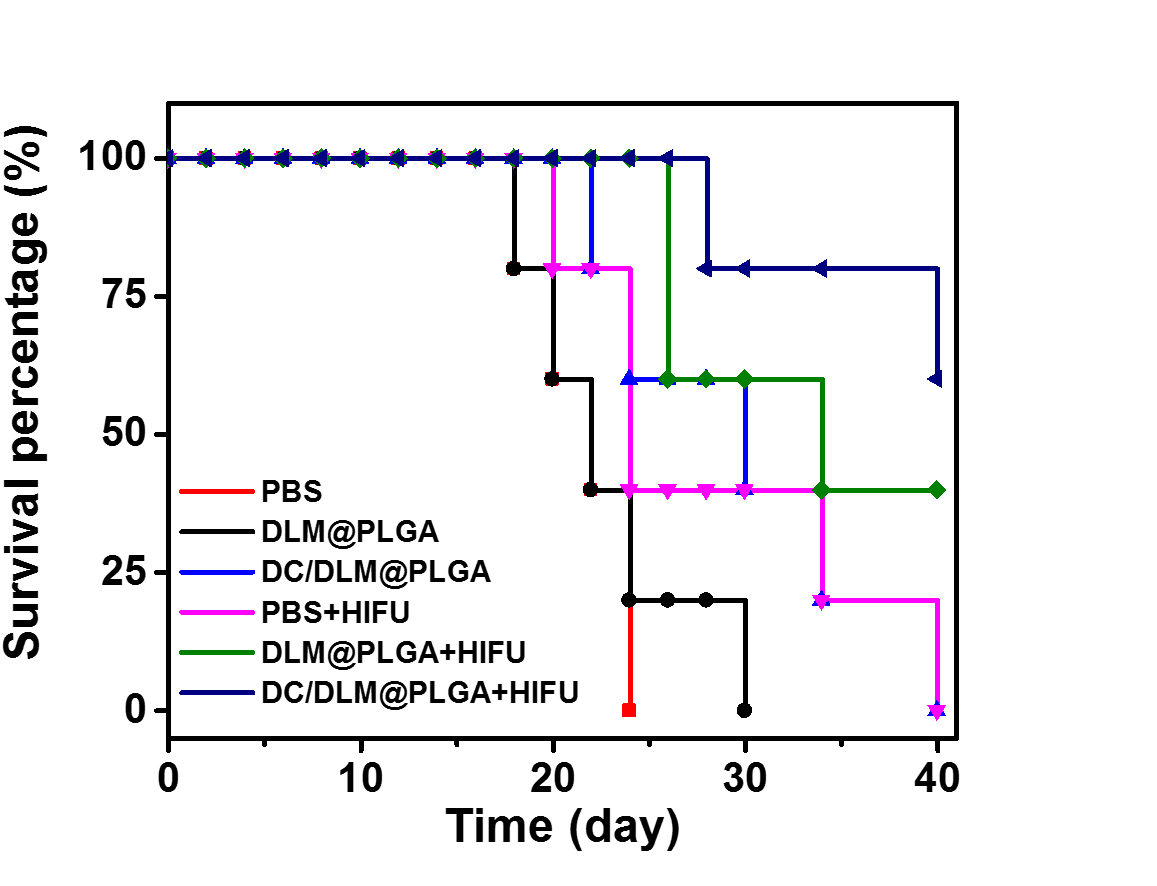


**Fig. S15.** Survival rate of the 4T1 tumor bearing-mice with various treatments as indicated (n=5).


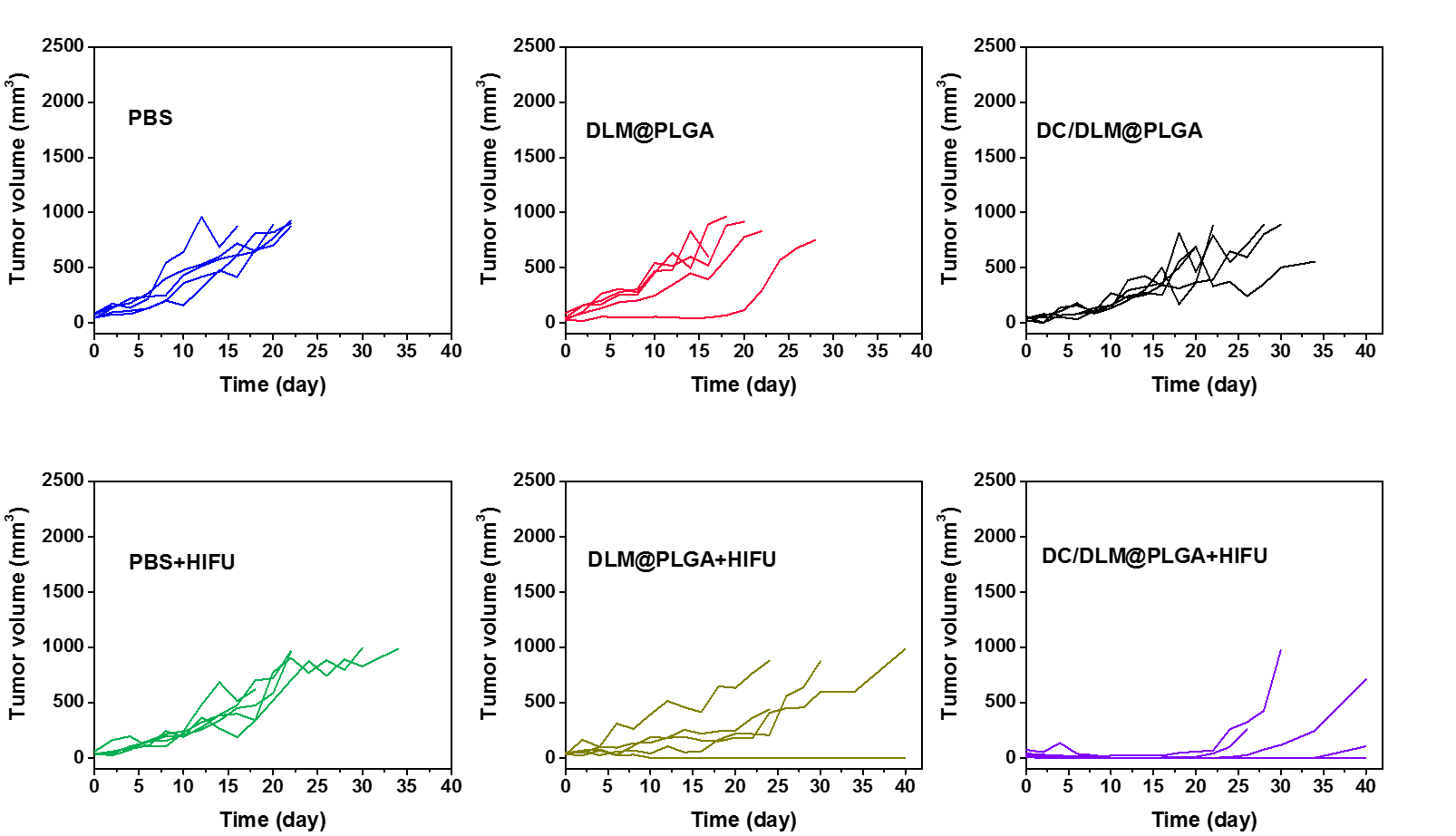


**Fig. S16.** Changes in tumor volume of 4T1 tumor bearing-mice of various treatments as indicated (n=5).
